# Ancient and Novel Small RNA Pathways Compensate for the Loss of piRNAs in Multiple Independent Nematode Lineages

**DOI:** 10.1371/journal.pbio.1002061

**Published:** 2015-02-10

**Authors:** Peter Sarkies, Murray E. Selkirk, John T. Jones, Vivian Blok, Thomas Boothby, Bob Goldstein, Ben Hanelt, Alex Ardila-Garcia, Naomi M. Fast, Phillip M. Schiffer, Christopher Kraus, Mark J. Taylor, Georgios Koutsovoulos, Mark L. Blaxter, Eric A. Miska

**Affiliations:** 1 MRC Clinical Sciences Centre, Imperial College London, London, United Kingdom; 2 Department of Life Sciences, Imperial College London, London, United Kingdom; 3 The James Hutton Institute, Invergowrie, Dundee, United Kingdom; 4 Department of Biology, University of North Carolina at Chapel Hill, Chapel Hill, North Carolina, United States of America; 5 Center for Evolutionary and Theoretical Immunology, Department of Biology, University of New Mexico, Albuquerque, New Mexico, United States of America; 6 Department of Botany, University of British Columbia, Vancouver, British Columbia, Canada; 7 Zoologisches Institut, Universität zu Köln, Cologne, NRW, Germany; 8 Molecular and Biochemical Parasitology, Liverpool School of Tropical Medicine, Liverpool, United Kingdom; 9 Institute of Evolutionary Biology, University of Edinburgh, Edinburgh, United Kingdom; 10 Wellcome Trust/Cancer Research UK Gurdon Institute, Cambridge, United Kingdom; University of Bath, UNITED KINGDOM

## Abstract

Small RNA pathways act at the front line of defence against transposable elements across the Eukaryota. In animals, Piwi interacting small RNAs (piRNAs) are a crucial arm of this defence. However, the evolutionary relationships among piRNAs and other small RNA pathways targeting transposable elements are poorly resolved. To address this question we sequenced small RNAs from multiple, diverse nematode species, producing the first phylum-wide analysis of how small RNA pathways evolve. Surprisingly, despite their prominence in *Caenorhabditis elegans* and closely related nematodes, piRNAs are absent in all other nematode lineages. We found that there are at least two evolutionarily distinct mechanisms that compensate for the absence of piRNAs, both involving RNA-dependent RNA polymerases (RdRPs). Whilst one pathway is unique to nematodes, the second involves Dicer-dependent RNA-directed DNA methylation, hitherto unknown in animals, and bears striking similarity to transposon-control mechanisms in fungi and plants. Our results highlight the rapid, context-dependent evolution of small RNA pathways and suggest piRNAs in animals may have replaced an ancient eukaryotic RNA-dependent RNA polymerase pathway to control transposable elements.

## Abstract 2

A survey of the nematode phylum reveals loss of the Piwi/piRNA pathway in several lineages, but RNA-dependent RNA polymerases control transposable elements in its absence.

## Author Summary

Transposable elements are segments of DNA that have the ability to copy themselves independently of the host genome and thus pose a severe threat to the integrity of the genome. Organisms have evolved mechanisms to restrict the spread of transposable elements, with small RNA molecules being one of the most important defense mechanisms. In animals, the predominant small RNA transposon-silencing mechanism is the piRNA pathway, which appears to be widely conserved. However, little is known about how small RNA pathways that target transposons evolve. In order to study this question we investigated small RNA pathways across the nematode phylum, using a well-studied model organism—the nematode *Caenorhabditis elegans*—as the starting point. Surprisingly we found that the piRNA pathway has been completely lost in all groups of nematodes bar those most closely related to *C*. *elegans*. This finding raises the intriguing question of how these nematodes are able to control transposable element mobilization without piRNAs. We discovered that there are other small RNA pathways that target transposable elements in these nematodes, employing RNA-dependent RNA polymerases in order to make small RNAs antisense to transposable elements. Intriguingly, the most ancient of these mechanisms, found in the most basal nematodes, is a Dicer-dependent RNA-directed DNA methylation pathway. This pathway shares strong similarity to transposon-silencing mechanisms in plants and fungi, suggesting that it might have been present in an ancient common ancestor of all eukaryotes. Our results highlight the rapid evolution of small RNA pathways and demonstrate the importance of examining molecular pathways in detail across a range of evolutionary distances.

## Introduction

Transposable elements are found in almost all eukaryotic genomes, and present a severe threat to the integrity of the germline and the survival of the species. Consequently, organisms have evolved robust pathways to silence the expression of transposable elements and restrict their spread [1–3]. Small (18–35 nucleotide [nt]) RNAs are amongst the most important of these pathways and several different transposon-control mechanisms involving small RNAs are found across the eukaryotic domain. In animals, transposon silencing is controlled by Piwi-interacting small RNAs (piRNAs), which associate with the conserved Piwi subfamily of Argonaute proteins and are essential for fertility in *Drosophila melanogaster*, *Danio rerio*, *Mus musculus*, and the nematode *C*. *elegans* [4,5]. Along with microRNAs, which associate with the Ago subfamily of Argonautes, piRNAs are widely conserved across the animal kingdom [4,6,7]. However, other small RNA pathways are restricted to specific phyla, and the evolutionary and functional relationships between them are unclear, particularly because the majority of information available relates to a few very distantly related model organisms. Thus to understand how small RNA pathways evolve, a range of organisms over a variety of different evolutionary distances need to be studied. To carry out such an analysis we chose to study their evolution across the phylum Nematoda.

The best understood nematode is the model organism *C*. *elegans*, in which extensive studies of small RNAs have been undertaken. *C*. *elegans* possesses several classes of small RNAs, most of which are conserved. In *C*. *elegans* as in other organisms microRNAs (miRNAs) are transcribed from individual genomic loci to form hairpins that are processed by the activity of Dicer to produce mature miRNAs. The sequences of many *C*. *elegans* miRNAs are highly conserved all the way to humans and they have important functions in regulating key developmental transitions [8]

The *C*. *elegans* genome encodes two members of the Piwi subfamily of Argonautes, PRG-1 and PRG-2. The *prg-2* gene is the result of a recent gene duplication and does not appear to function directly in the piRNA pathway, but *prg-1* encodes a functional protein, which is expressed in the germline and binds to piRNAs [9,10]. However, in contrast to the high conservation of miRNAs and miRNA-processing, *C*. *elegans* piRNAs have some important differences to piRNAs in other animals. In *C*. *elegans* the 5′ U bias common to most animal piRNAs is conserved; however, they are they are only 21 nt long as opposed to the 26–30 nt more common in *M*. *musculus* and *D*. *melanogaster* [9,10]. In addition, piRNAs are produced from individual loci that are transcribed to produce short (26–30 nt) precursors that are processed to give rise to mature piRNAs [11,12], as opposed to the long piRNA precursor transcripts produced in *M*. *musculus* and *D*. *melanogaster* that are processed to give rise to multiple piRNAs per genomic locus [7,13]. The majority of piRNA loci in *C*. *elegans* are associated with an upstream sequence motif [9,10,14].

*C*. *elegans* piRNAs also differ from *D*. *melanogaster* and *M*. *musculus* piRNAs owing to their different mechanism of target silencing. piRNA-mediated silencing does not involve the direct cleavage of targets by the Slicer endonuclease domain of PRG-1. Instead, piRNAs silence their targets by initiating the synthesis of an abundant class of small interfering RNAs (siRNAs) through an RNA-dependent RNA polymerase (RdRP) [10,15]. These siRNAs align predominantly antisense to targets, are ~22 nt, and start with a guanine (G), thus are also referred to as 22G-RNAs [16,17]. Importantly, because each 22G-RNA is produced by a RdRP, they carry a 5′ triphosphate, whilst both piRNAs and miRNAs possess a 5′ monophosphate [16,17]. The *C*. *elegans* RdRPs RRF-1 and EGO-1 are required for 22G-RNA biogenesis, with the RRF-2 RdRP being dispensable [18]. The fourth *C*. *elegans* RdRP, RRF-3 is required instead for the production of another class of small RNAs, the 26G-RNAs, which have a 5′ monophosphate [19]. It remains unclear whether RRF-3′s catalytic activity is required for 26G-RNA production. 22G-RNAs associate with multiple “worm”-specific Argonaute proteins (WAGOs) [5] to bring about target silencing, and in addition to being produced downstream of piRNA targeting, 22G-RNAs are also produced downstream of target recognition by other classes of endogenous small RNAs and RNA interference induced by exposure to double-stranded RNA (dsRNA) [20].

Despite divergence in biogenesis and silencing mechanisms, *C*. *elegans* piRNAs have a similar function to those in other organisms, as they target transposable elements for silencing [10,15]. Additionally, *C*. *elegans prg-1* mutants show fertility defects [5], and become sterile over many generations [21], meaning that an important role for the piRNA pathway in protecting the function of the germline is conserved across animal species. Thus the *C*. *elegans* piRNA pathway represents an interesting example of where a conserved central core (the Piwi/piRNA complex) has acquired different upstream and downstream components whilst retaining its ancestral function.

**Fig 1:**
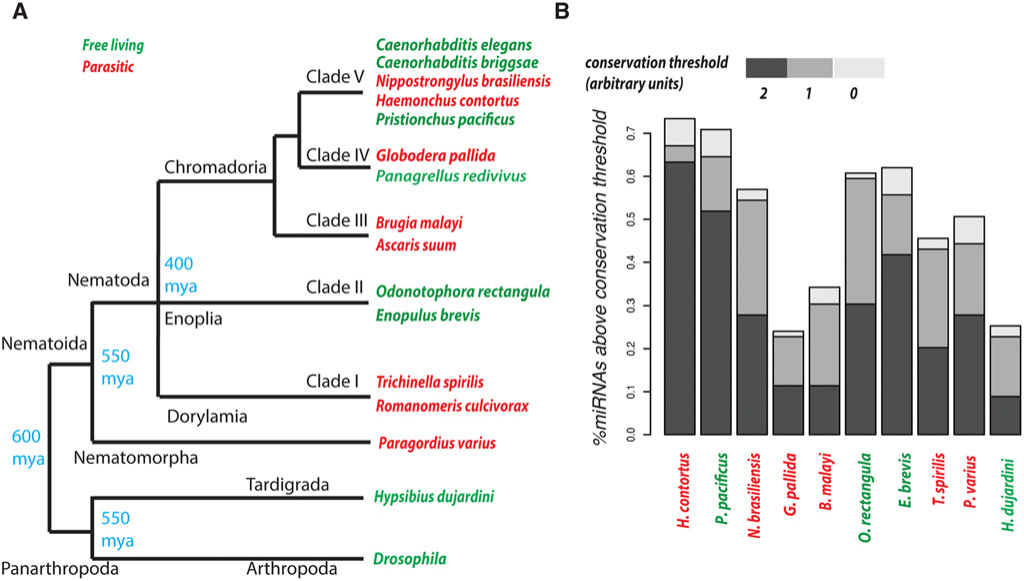
Conservation of miRNAs in the phylum Nematoda. (A) Cladogram of the phylogenetic systematics of Nematoda, using the clades I–V as defined by Blaxter and colleagues [22]. Numbers in blue represent approximate divergence times for key nodes [23]. Red and green represent parasitic and free-living species respectively. (B) Percentage of *C*. *elegans* miRNAs conserved in nematode species coloured according to using three different stringency cutoffs (see S1 Text for detailed description).

In order to gain further insight into how piRNAs evolve in the context of other small RNA pathways, we used *C*. *elegans* as a basis to guide an examination of small RNAs and the proteins that bind to them across the known diversity of the phylum Nematoda (Fig. 1A). Our analysis reveals that, surprisingly, piRNAs have been lost several times independently across the phylum. Instead, we find that in the Chromadoria group of nematodes (clades III–V), 22G-RNAs produced by RNA dependent RNA polymerase operate in the absence of piRNAs to target transposable elements. In the Dorylamia group of nematodes, more distant to *C*. *elegans*, (clades I/II), small RNAs targeting transposons are produced by a different RNA dependent RNA polymerase pathway, in this case acting processively to generate dsRNA that is then processed by Dicer into small RNAs. This pathway also involves DNA methylation of transposable elements thus displays similarity to the RNA directed DNA methylation pathway found in plants and fungi. Our results provide a clear example of the rapid diversification of molecular pathways involved in silencing repetitive elements but at the same time identify hitherto unknown conservation at the core of the eukaryotic small RNA machinery.

## Results and Discussion

**Fig 2:**
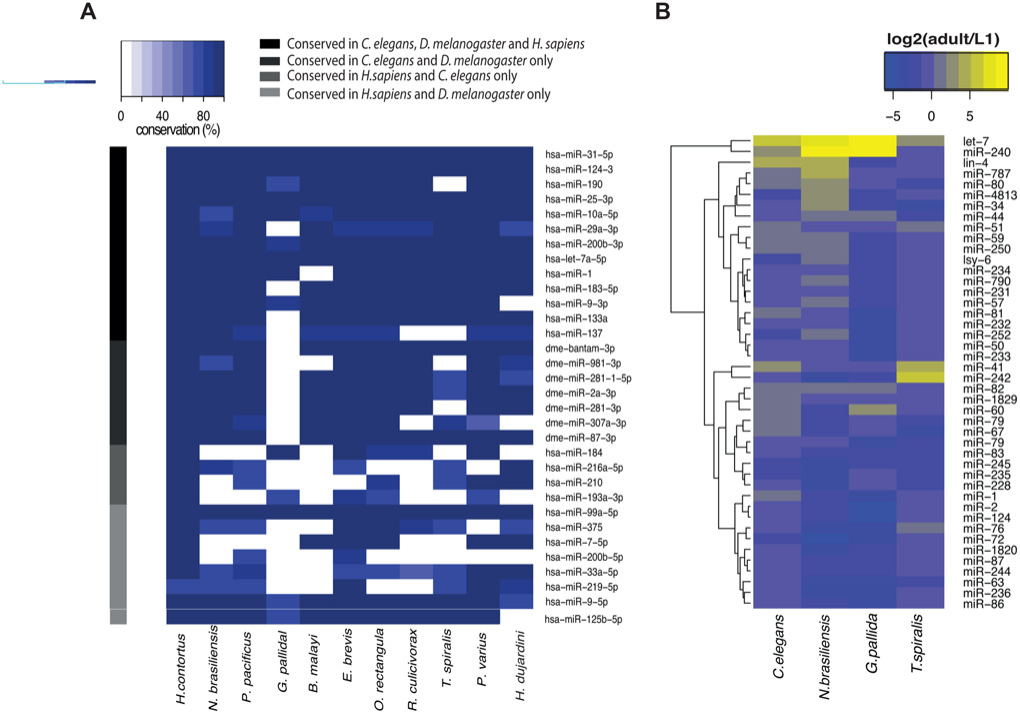
miRNA families and expression in the phylum Nematoda. (A) Conservation of conserved miRNA families, grouped by seed in nematodes. The family is named after the most abundant *H*. *sapiens* family member, or the human member if no *H*. *sapiens* member exists. The colour bar indicates conservation of a miRNA in *C*. *elegans*, *D*. *melanogaster*, *H*. *sapiens* or a combination thereof. (B) The developmental profile of miRNAs is conserved across Nematoda. All *C*. *elegans* miRNAs present in all of the species profiled are shown.

To investigate small RNA pathway evolution, we sequenced small RNAs from 11 species across the phylum Nematoda, and analysed previously published data from two more, spanning the known diversity (Fig. 1A; S1 Text). We selected at least one species from each of the five clades (I–V; *C*. *elegans* is in clade V) [22]. Our sample included parasites of animals and plants, and free-living nematodes, and included two representatives of the neglected clade II marine species. Additionally, where possible, we collected samples from at least two life cycle stages (S1 Text), always including adults, as some small RNA pathways are enriched in or exclusive to the germline in *C*. *elegans* [9,10]. We first analysed small RNAs with a 5′ monophosphate, which in *C*. *elegans* include miRNAs and piRNAs (S1 Fig.). To assess miRNA conservation we used the annotated *C*. *elegans* miRNAs as a reference and calculated a conservation score based on sequence conservation and relative expression levels (see Materials and Methods). By this measure all nematode species showed conservation of at least 20% of *C*. *elegans* miRNAs (Fig. 1B), with the proportion of miRNAs conserved rising with decreasing phylogenetic distance to *C*. *elegans* (Spearman’s Rho = 0.47; *p* = 0.02). We focussed on miRNA families that are conserved in at least two out of *C*. *elegans*, *D*. *melanogaste*r, and *Homo sapiens* as likely ancestral bilaterian miRNAs. The majority were conserved across all the species we examined, and only two of these miRNAs were apparently lost in all nematodes profiled; however, there were isolated examples of species-specific losses of miRNAs, suggesting that their conservation amongst bilaterians is not universal (Fig. 2A). Additionally, we compared small RNA levels for miRNAs at early larval (L1 or L2) and adult stages for *C*. *elegans*, *Trichinella spiralis* (clade I), *Globodera pallida* (clade IV), and *Nippostrongylus brasiliensis* (clade V), as the life cycles of these species permitted collection of the required life stages. Broadly, these nematodes all showed similar developmental miRNA expression dynamics to *C*. *elegans* with correlation between expression changes in *C*. *elegans* and other nematodes better for more closely related nematodes (Figs. 2B and S2). Taken together, these data show that most miRNA sequences and their developmental regulation are highly conserved across the Nematoda.

**Fig 3:**
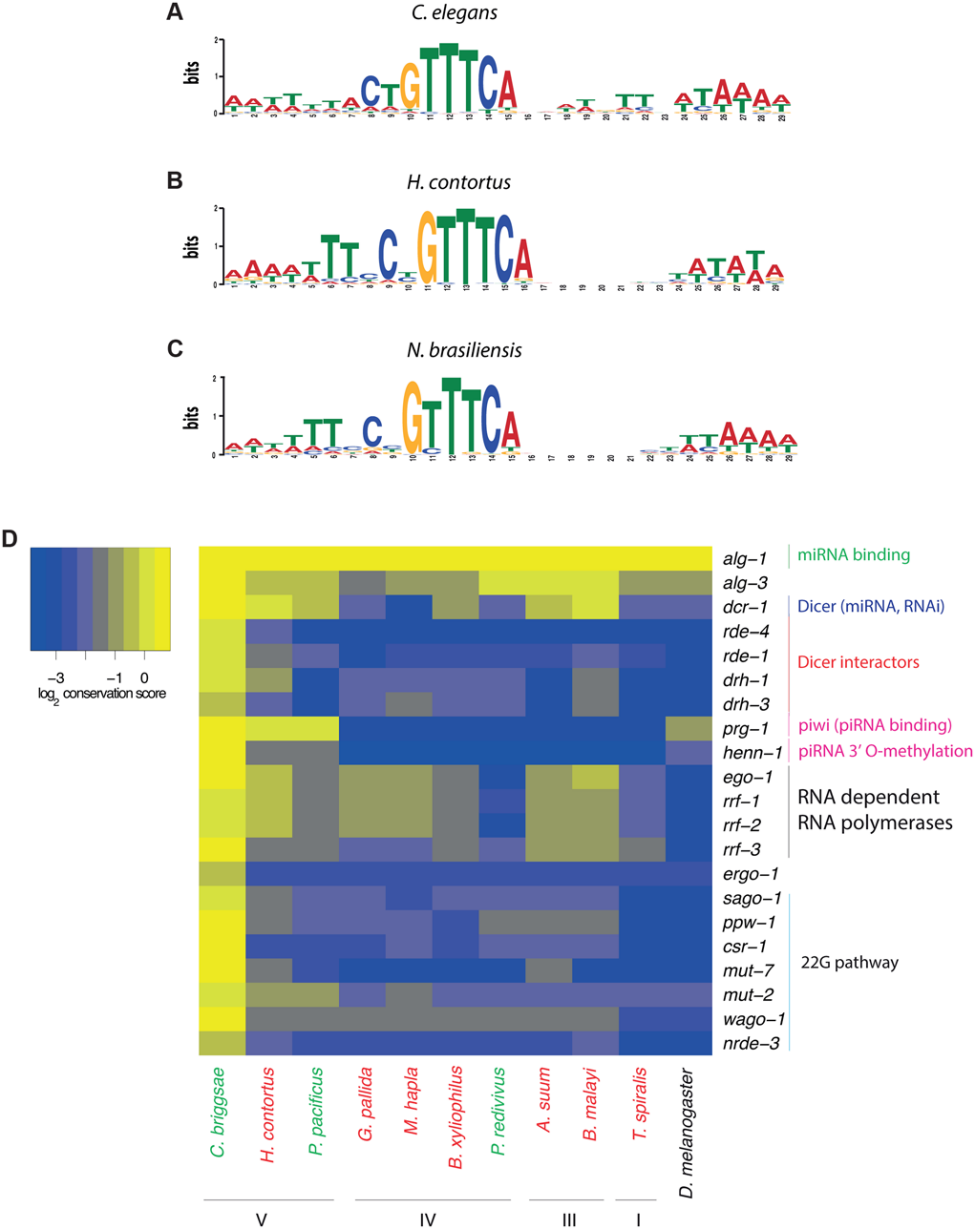
Loss of the piRNA pathway in nematodes outside of clade V. (A–C) Sequence motifs were *de novo* predicted in the upstream regions of aligned 21U-RNA sequences in clade V nematodes: (A) *C*. *elegans*; (B) *H*. *contortus*; (C) *N*. *brasiliensis*. The motifs are presented as sequence logos generated using the MEME program [27]. The upstream sequences for *C*. *elegans* were taken from [9]. The upstream sequences for *H*. *contortus* and *N*. *brasiliensis* 21U-RNAs are in the S4 Data. (D) Conservation of selected *C*. *elegans* small RNA pathway proteins. Conservation is calculated as the logarithm of the score of the best blast hit in bits normalized to the length of the protein (see S1 Text).

**Fig 4:**
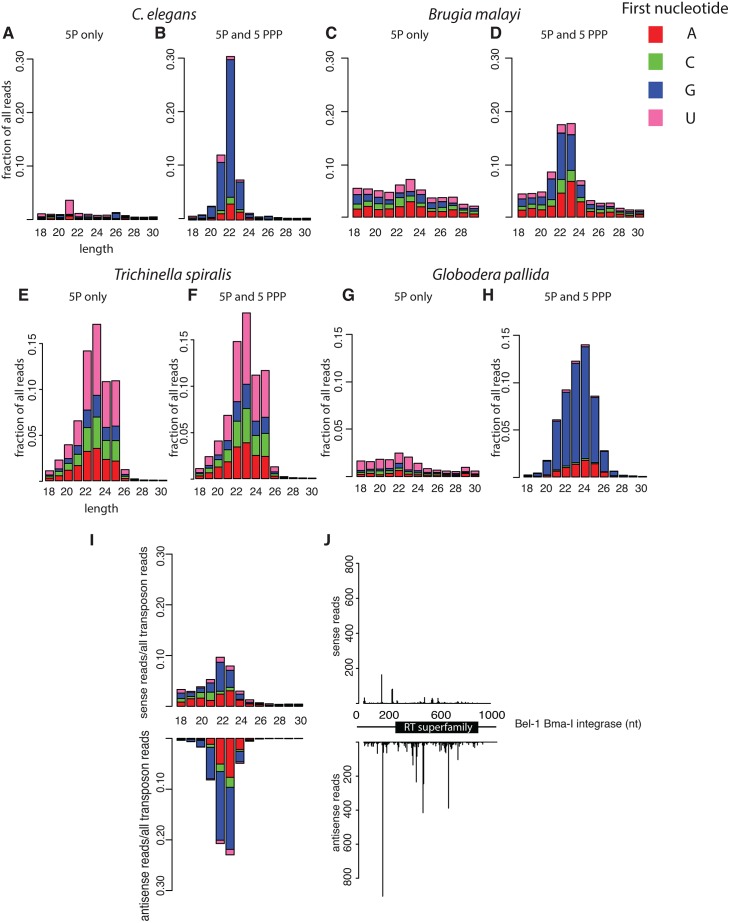
siRNAs combat transposons across the phylum Nematoda. (A–H) Non-processive RdRP activity evolved in the ancestor of clade III–V nematodes. Comparison between libraries prepared from 5′ monophosphate only and 5′ monophosphate and 5′ triphosphate material demonstrates that 5′ triphosphate small RNAs are present in *C*. *elegans* (A, B), *G*. *pallida* (G, H), and *B*. *malayi* (C, D) but not found in *T*. *spiralis* (E, F). (I) 22G-5′ triphosphate small RNAs target transposons in *B*. *malayi*. (J) An example *B*. *malayi* transposon showing the distribution of small RNAs across the predicted transposon sequence. The transposon shown is a member of the BEL/PAO retrotransposon family. Complete Repeatmasker annotations of *B*. *malayi* are in S4 Data.

We next investigated piRNAs. In *C*. *elegans*, piRNAs are 21 nt long and start with a uracil (U) also referred to as 21U-RNAs [9,10,14]. Most piRNAs in *C*. *elegans* are clustered in the genome and are associated with a defined upstream sequence motif [9,10]. Previous analysis has shown that similar upstream motifs are found upstream of 21U-RNAs in other *Caenorhabditis* nematodes and in *Pristionchus pacificus* [1,3]. Since the motif may have diverged in other nematodes we aligned small RNAs to their source genomes and searched for motifs upstream of putative piRNA loci. Using this approach we were able to recover a motif with strong similarity to the *C*. *elegans* core motif upstream of 21U-RNAs in *Haemonchus contortus* [24] (clade V) and *N*. *brasiliensis* (Fig. 3A–3C). Thus the 21U-RNAs and the *C*. *elegans* core motif are highly conserved across the clade V nematodes. However, we were unable to identify a motif for any nematode species outside of clade V. This is unlikely to be due to poor genome assembly as the genome of *N*. *brasiliensis* is the least complete of any of the genomes in our study (S1 Text). Moreover, when predicted miRNAs were excluded from the analysis, we did not detect small RNAs of any size with a 5′ U bias in non-clade V species (Figs. 4 and S4). Thus we did not detect 21U-RNAs or 25–32 nt long piRNAs in nematodes outside clade V. Consistent with our analysis, piRNAs were previously reported to be absent in the parasitic nematode *Ascaris suum* (clade III) [25] and the free-living nematode *Panagrellus redivivus* (clade IV) [26]. Given the topology of the phylogenetic tree (Fig. 1A), these data suggest that the piRNA pathway has been lost independently in multiple nematode lineages.

To confirm the loss of piRNAs, we explored the conservation of proteins involved in the RNA interference (RNAi) pathway, as defined in *C*. *elegans* (Fig. 3D). We found high conservation of the proteins involved in the miRNA pathway as well as some proteins known to be involved in other endogenous small RNA pathways (see below). However the *C*. *elegans* Piwi orthologue PRG-1 was absent from all non-clade V nematodes [28] despite being highly conserved in *D*. *melanogaster* (Fig. 3D). Strikingly, the only other protein showing this pattern of conservation was HENN-1 (Fig. 3D), which stabilises piRNAs by adding a 2′ O methyl at the 3′ end and is thus an important component of the piRNA pathway in animals including *C*. *elegans* [29–33]. Furthermore, we also found no PRG-1 orthologue in other clade III and IV nematodes with genome assemblies, nor analysing draft transcriptome data from *E*. *brevis* (clade II) (S1 Text). This distribution of conservation is unlikely to be due to a sampling error due to incomplete genome assemblies because such a distribution of conservation, placing *D*. *melanogaster* closer to *C*. *elegans* than to any non-clade V nematodes was very unusual within the *C*. *elegans* proteome (estimated Jack-knife *p* < 10^4^: see Materials and Methods). Moreover, this loss is unlikely to be an effect of rapid evolution of PRG-1 because it evolves more slowly relative to the median rate of all *C*. *elegans* proteins in all the species in which we detect it (S5A Fig.). To test this hypothesis further, we simulated evolving PRG-1 to the distance between PRG-1 and *D*. *melanogaster* Piwi 1,000 times and spiked the simulated protein into the *T*. *spiralis* gene set. In 1,000 simulations the maximum e-value we observed, corresponding to the weakest hit, between the simulated protein and PRG-1 was still 10^10^ lower than the best hit to PRG-1 in the true *T*. *spiralis* set (S5B Fig.).

**Fig 5:**
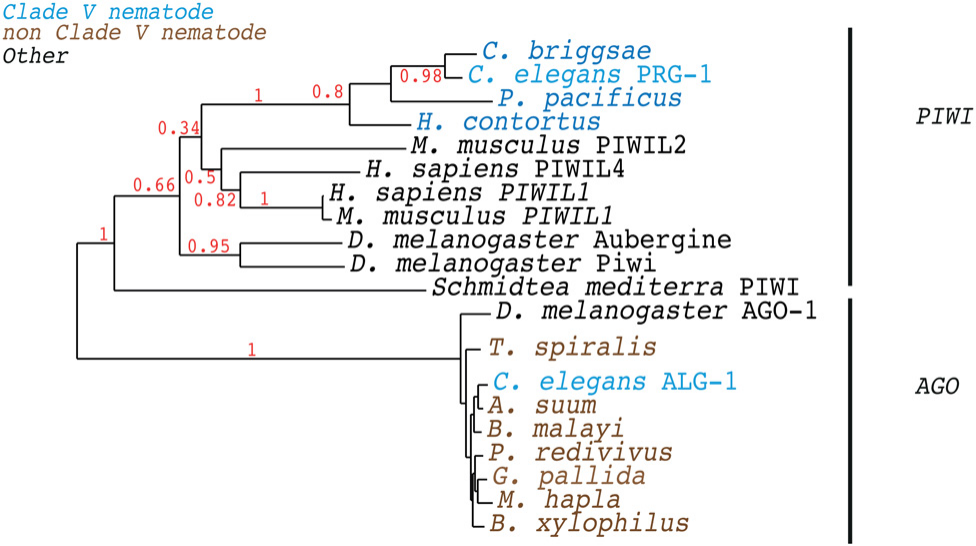
Loss of the Piwi protein in nematodes outside of clade V. Maximum likelihood phylogenetic tree of the best homologues of *D*. *melanogaster* Piwi found in the analysed nematode genomes. We included a subset of known Piwi proteins and Ago proteins from other organisms for comparison. The alignment is in S1 Data. Bootstrap branch support values from 100 bootstraps are shown. The tree file is in S4 Data.

In order to test whether an independent approach could reproduce the loss of PRG-1 in non-clade V nematodes we used phmmer to find the highest scoring homologue of *D*. *melanogaster* Piwi in all the nematode species we tested. We then constructed a maximum-likelihood phylogenetic tree of these proteins, spiking in the known Piwi proteins from mammals as well as *C*. *elegans* ALG-1 and *D*. *melanogaster* AGO as examples of members of the Ago subfamily, responsible for Dicer-dependent small RNA binding [6]. Whilst the best homologues to Piwi in every clade V nematode clustered with the other Piwi proteins, the best homologues of Piwi in every non-clade V nematode were clearly members of the Ago subfamily (Fig. 5). Taken together these data strongly support the loss of PRG-1, and with it the piRNAs that depend on Piwi proteins for their stability, independently in several nematode lineages outside of clade V.

As the repeated loss of piRNAs in such a speciose phylum was unexpected, we decided to examine other related ecdysozoan phyla. We first profiled small RNAs in tardigrades, which are Panarthropods, and thus more closely related to *D*. *melanogaster* than to nematodes [34]. We identified small RNAs in the tardigrade *Hypsibius dujardini* showing a strong 5′ U bias and with a modal length of 26 nt (S3A Fig.). Moreover, we identified a Piwi homologue from draft transcriptome data, suggesting that these small RNAs could be piRNAs (S1 Text). *H*. *dujardini* piRNAs were clustered on the genome, and showed a signature suggestive of ping-pong amplification as characterized in *D*. *melanogaster* [13], whereby the tenth nucleotide of overlapping transposons shows a bias towards adenine (A) (S3B Fig.). piRNAs in the tardigrade are thus more similar to those of *D*. *melanogaster* than to *C*. *elegans*.

We next examined *Paragordius varius*, member of the phylum Nematomorpha, which is the sister phylum of Nematoda [34]. Interestingly, although we clearly identified miRNAs in *P*. *varius*, including many that are widely conserved in nematodes (Fig. 2A), we were unable to identify longer piRNAs similar to those seen in tardigrades (S3C Fig.). Thus we concluded that the longer 25–32 nt piRNAs found in many animal phyla may have been lost in the common ancestor of Nematoda and Nematomorpha. Consistently, we were unable to identify a Piwi orthologue analysing draft transcriptome data from *P*. *varius* (S1 Text).

Given the essential role of piRNAs in targeting transposable elements for silencing, we wondered whether nematodes that lack piRNAs might use other small RNA pathway to target transposable elements. In *C*. *elegans*, piRNAs act upstream of the generation of 22G-RNAs by RNA dependent RNA polymerase [10]. However, 22G-RNA-mediated silencing can in some cases persist for some time independently of piRNA activity and can also compensate for the absence of piRNAs in some circumstances [21]. We therefore tested whether 22G-RNAs are able to compensate for the absence of piRNAs in nematodes outside of clade V. Importantly, this class of RdRP-derived small RNAs retain their 5′ triphosphate [16,17] allowing us to use comparison between small RNA sequencing of 5′ monophosphate only species with 5′ triphosphate and 5′ monophosphate containing species (S1 Fig.) to detect 5′ triphosphorylated small RNAs with a 5′ G in *Brugia malayi* (clade III) and *G*. *pallida* (clade IV). These small RNAs had a modal length of 22 nt in *B*. *malayi* and 22–26 nt in *G*. *pallida* (Fig. 4C, 4D, 4G, and 4H). However, we did not detect any 5′ triphosphorylated small RNAs in *T*. *spiralis* (clade I) (Fig. 4E and 4F), *Romanomeris culicivorax* (clade I), *Enoplus brevis* (clade II), or *Odontophora rectangula* (clade II) (S4 Fig.). This suggests that RdRP-derived 22G-RNAs evolved in the last common ancestor of nematode clades III–V.

**Fig 6:**
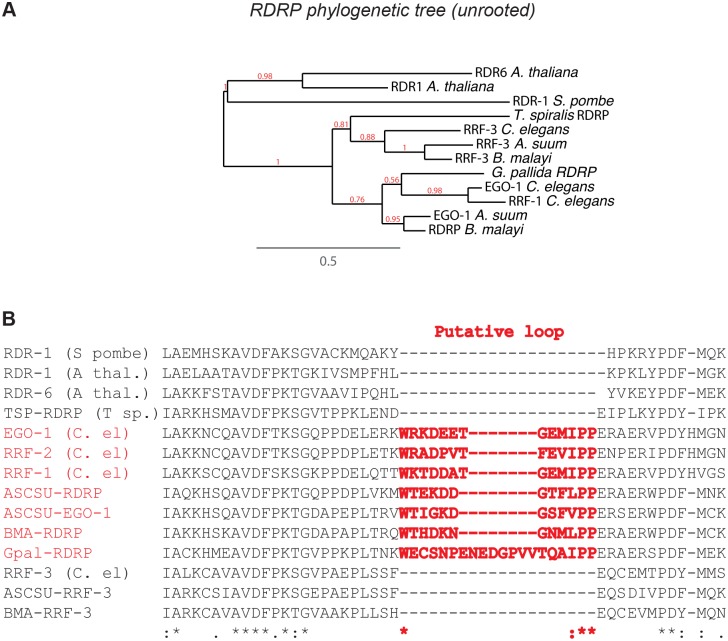
RNA dependent RNA polymerase sequence analysis. (A) Unrooted phylogenetic tree of RNA dependent RNA polymerase sequences from curated nematode genomes, *Arabidopsis thaliana*, and *S*. *pombe*. Whilst nematode RNA dependent RNA polymerases are a distinct class, RRF-3 and RRF-1/RRF-2/EGO-1 define separate subgroups and only the RRF-3 subgroup is conserved in *T*. *spiralis*. Branch support values from 100 bootstraps are shown in red at each bifurcation. The tree file is in S4 Data. (B) A portion of the multiple sequence alignment of RdRP (Muscle) where a conserved, proline-rich loop is inserted in RRF-1/RRF-2/EGO-1 family polymerases, not present in either RRF-3 family RdRPs or in plant or fungal RdRPs. The full sequence alignment is in S2 Data.

To examine this analysis further we examined the conservation of RdRPs in nematodes. In *C*. *elegans*, the RdRPs RRF-1, EGO-1, and RRF-2 generate 22G-RNAs whereas the RdRP RRF-3 is required for a separate small RNA pathway involving Dicer [35]. We identified RdRPs in all nematode clades. However RRF-1, RRF-2, and EGO-1 were only found in clades III–V (Fig. 3D; S1 Text), whilst RRF-3-like genes were found in all clades (Fig. 3D), including clade II (S1 Text). Thus the RRF-1 RdRP family likely arose in the last common ancestor of clades III–V. To investigate this further we performed multiple sequence alignment and phylogenetic analysis of eukaryotic RdRPs (Fig. 6). This analysis suggested that nematode RNA dependent RNA polymerases fall into two groups, the RRF-3 family RNA dependent RNA polymerases, conserved across the whole nematode phylum, and the RRF-1/EGO-1 family that is only conserved in clades III–V (Fig. 6A; S1 Text). Interestingly the RRF-1/EGO-1 family can be distinguished from RRF-3 on the basis of a conserved insertion, containing two proline residues and a tryptophan residue, which is not present in RRF-3 or any RdRPs from other organisms (Fig. 6B). In viruses, a key difference between de novo initiating RdRPs and those that initiate in a primer-dependent manner is the presence of an extra loop in de novo polymerases, often containing aromatic residues, which enables the initiating G nucleotide to be fixed in the active site despite weak interactions with the template [36]. Although eukaryotic RdRPs are very distantly related to viral RdRPs, and possess different catalytically active residues [37], there are some similarities between the overall architecture of the only eukaryotic RdRP with a solved structure, QDE-1 from *Neospora crassa*, and viral RdRPs [38]. In vitro QDE-1 uses looped-back RNA from the template as a primer [39,40], although the enzyme may also initiate de novo at the 3′ end of certain templates [39]. It is therefore plausible that, analogous to viral RdRPs, the conserved extra loop in RRF-1 family RdRPs is important for de novo initiation, whilst the more ancient RRF-3 family polymerases utilise primer-dependent initiation similarly to QDE-1. This would be consistent with the different products of the two groups of nematode RdRPs: short, 5′ triphosphorylated G-RNAs arise from the activity of the de novo initiating RRF-1/EGO-1 family polymerases whilst longer RNAs are made by the RRF-3 polymerases (Table 1).

**Table 1:** Summary of the presence of key components of the *C*. *elegans* small RNA pathway in different nematode clades and *D*. *melanogaster*.

Group	RdRP (RRF-3 type)	RdRP (EGO-1 type)	PRG-1/PIWI
D. melanogaster	No	No	Yes
Clade I	Yes	No	No
Clade II	Yes	No	No
Clade III	Yes	Yes	No
Clade IV	Yes	Yes	No
Clade V	Yes	Yes	Yes

Having established that 22G-RNA RdRP products are conserved in clades III–V we tested whether they could be important in defence against transposable elements in nematodes lacking piRNAs. We identified potential transposable element sequences in the nematode genomes with RepeatMasker, and aligned small RNAs to them to identify potential siRNAs. *B*. *malayi* and *G*. *pallida* both had abundant 5′ triphosphate RNAs aligning to transposons (Fig. 4I and 4J; S6 Fig.). There were more 22G siRNA sequences mapping antisense than sense (for both: *p* < 2.2e−16, Chi-squared test against a uniform distribution). In *B*. *malayi* the antisense bias was greater for 22G-RNAs than for non 22G-RNA sequences, whilst in *G*. *pallida* the antisense bias was seen for 20–26 nt long 5′ triphosphorylated G-RNAs (*p* = 3.4e−7 and *p* = 7.7e−4 respectively, Chi-squared test with Yates continuity correction). This finding suggests that transposon silencing is mediated by RdRPs in species other than *C*. *elegans*, even though these species lack piRNAs.

We wondered what might initiate the formation of RdRP siRNAs against transposons in the absence of piRNAs. Interestingly, we detected small quantities of 22–23 nt 5′ monophosphate RNAs aligning to both strands of transposons in *B*. *malayi* and *G*. *pallida* (Figs. 4 and S6). These are likely siRNAs generated by Dicer acting on dsRNA and may therefore act to recruit RdRP activity to transposons without the activity of piRNAs (S7 Fig.). It is not clear why *C*. *elegans* and other clade V nematodes have retained the piRNA pathway in addition to the 22G-RNA pathway. PRG-1 has functions independent of initiating 22G-RNA mediated silencing [21], thus one possibility is that clade V-specific transposable elements exist that cannot be silenced by 22G-RNAs but require PRG-1 for their control. Loss of these elements in other lineages might have enabled subsequent loss of the Piwi protein.

**Fig 7:**
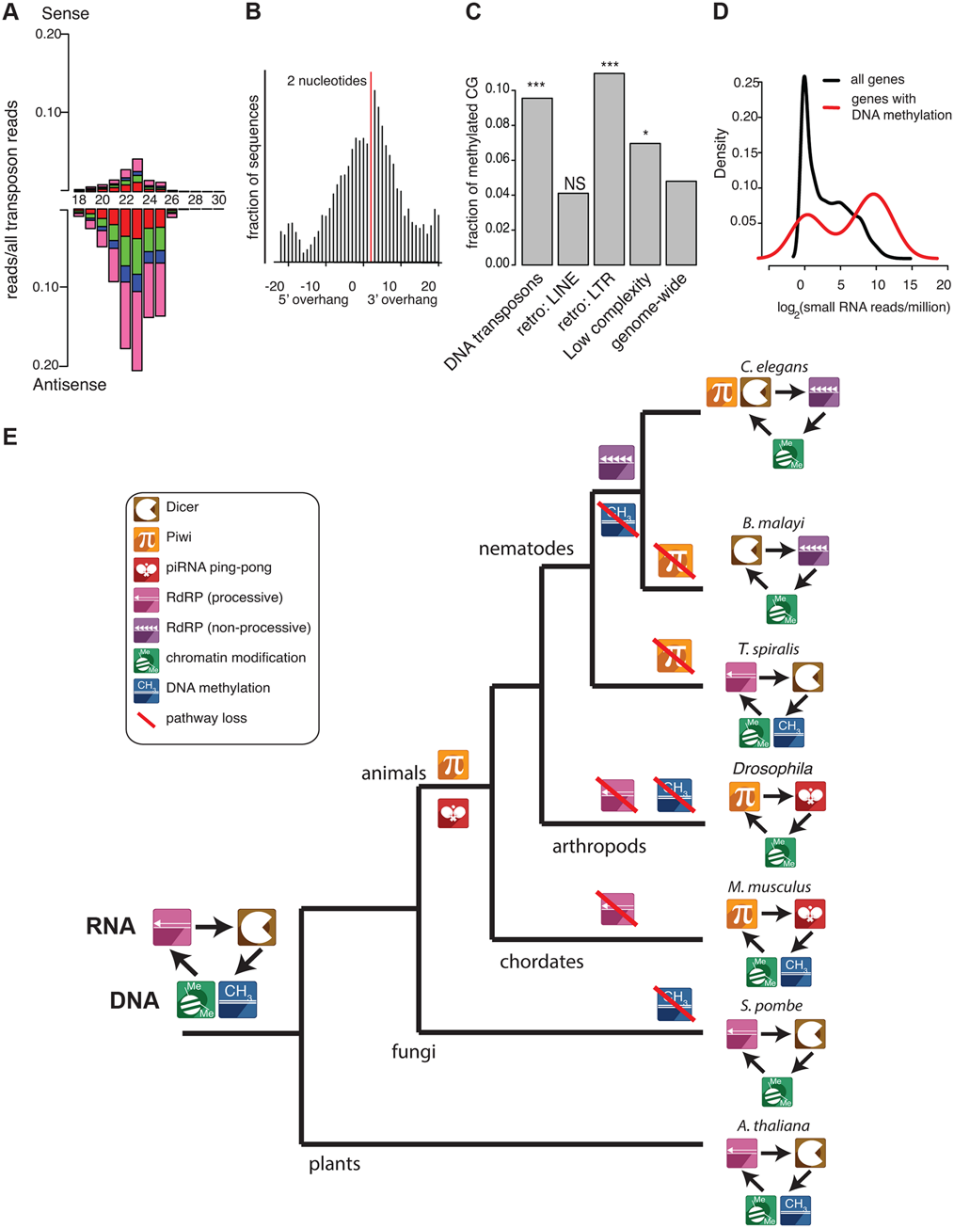
Conservation of eukaryotic transposon-defence mechanisms. (A) *T*. *spiralis* endo siRNAs align predominantly antisense to transposon sequence. (B) Anti-transposon endo siRNAs in *T*. *spiralis* have the characteristic 2 nt overhang of Dicer products. (C) CpG DNA methylation in *T*. *spiralis* is enriched at LTR retrotransposons and DNA transposons relative to genome-wide CG DNA methylation levels. Categories shown are as defined by Repeatmasker. ***chi-squared *p* < 1e−40; *chi-squared *p* < 1e−5. *T*. *spiralis* Repeatmasker annotations are in the supporting information. DNA methylation data for the analysis was taken from [41]. (D) Evidence for RNA directed DNA methylation in *T*. *spiralis*. The density plot shows small RNA reads for all genes (black) and genes with DNA methylation (red). A shift towards higher levels of small RNA reads is seen for DNA methylated genes. (E) A model for the evolution of transposon silencing pathways. Expression of transposable elements is recognized at the level of RNA through structural or sequence features. Consequently, transposable elements are silenced post-transcriptionally through cleavage (RNA) or transcriptionally through histone modification and/or DNA methylation (DNA). This interplay between the RNA and DNA level through different pathways is shown here. Symbols indicate pathways used for defence against transposons in a species, not conservation of individual protein factors. Note that RdRPs can act upstream or downstream of Dicer. Branch lengths are for illustration only.

*T*. *spiralis* has neither piRNAs nor 5′ triphosphate small RNAs. Nevertheless, aligning small RNAs from *T*. *spiralis* to transposon sequences showed abundant 23–25 nt RNAs enriched antisense to transposons (Fig. 7A; *p* < 2e−16, Chi-squared test against a uniform distribution). Several features of these sequences suggest that they are of an evolutionary distinct origin to the piRNAs found in either *C*. *elegans* or *D*. *melanogaster*. First, piRNAs are made by Dicer-independent mechanisms [7]. However, overlapping sense-antisense pairs of *T*. *spiralis* small RNAs showed a two nt 3′ overhang consistent with their being the product of Dicer activity on dsRNA (Figs. 7B and S1). Second, piRNAs in *C*. *elegans* and *D*. *melanogaster* are produced by transcription from clusters containing exceptionally high numbers of piRNA sequences [13,14]; *T*. *spiralis* 23–25 nt small RNAs, however, are distributed more evenly throughout the genome (*p* < 1e−7 to *D*. *melanogaster*, *p* = 0.01 to *C*. *elegans*, Kolmogorov-Smirnov test for different distributions), such that clusters with >10 times the mean density of small RNAs genome-wide are virtually absent (S8 Fig.). Third, piRNAs are typically enriched for 2′ O-methylation. We tested for this modification using protection against sodium periodate and whilst we readily detected specific protection of *C*. *elegans* 21U-RNAs, *T*. *spiralis* 23–25 nt small RNAs were not protected by the treatment (S8 Fig.), consistent with our finding that the HENN-1 protein responsible for 2′ O-methylation of piRNAs is not conserved in *T*. *spiralis* (Fig. 3D).

Given that the *T*. *spiralis* transposon-silencing small RNAs were produced by Dicer, and that RNA dependent RNA polymerase is conserved in *T*. *spiralis*, we wondered whether RdRP could produce dsRNA as a substrate for Dicer using the transposon mRNA as a template (S6 Fig.). Such a pathway is found in plants and fungi, and acts upstream of DNA methylation and/or histone modification to silence transposons, a process known as RNA-directed DNA methylation [41–43]. While absent in *C*. *elegans* and related nematodes, DNA methylation is found in *T*. *spiralis* [44] and the DNA methylation machinery is present in *R*. *culicivorax* [45]. We analysed CG methylation using genome-wide bisulfite sequencing of *T*. *spiralis* adults [44] and found a highly significant increase in meCpG density in LTR retrotransposons and DNA transposons compared to genome-wide meCpG (Fig. 7C). Moreover we observed a striking correlation between methylated regions of the genome and enrichment for small RNAs (Fig. 7D), implying that RNA-directed DNA methylation may occur in *T*. *spiralis*. Furthermore one of the three Dicer paralogues in *T*. *spiralis* contains a high-scoring bipartite nuclear localization motif (S3 Data). *Schizosaccharomyces pombe* Dicer also has a bipartite nuclear localization motif and enzymes involved in plant RNA-directed DNA methylation are nuclear localized [46,47]. Thus we propose that RNA-directed DNA methylation mediated by RdRPs and nuclear Dicer acts to silence transposon sequences in *T*. *spiralis*.

We suggest that RNA-directed DNA methylation involving Dicer and RdRP activity is an ancestral transposon silencing mechanism for eukaryotes (Fig. 7E). This system provides target recognition, amplification of small RNAs, and transcriptional repression thus ensuring robust transposon silencing. During the evolution of animals, this central module diversified to utilise piRNAs for target recognition. In some cases, as in mammals or *D*. *melanogaster*, piRNAs can operate independently of RdRP activity by using a different amplification strategy (the ping-pong mechanism). In surveying the diversity of small RNAs within Nematoda, we found evidence of both the ancestral Dicer/RdRP pathway and the piRNA pathway. In addition a unique non-processive RdRP activity evolved, responsible for generation of a novel class of small RNAs—the 22G-RNAs. We predict that similar diversity is likely to be evident across other animal phyla—indeed, evidence exists for RdRPs in some arthropods [37]—underlining the need to survey wide groups of organisms beyond a few well characterised model systems in order to understand how molecular pathways have evolved.

## Materials and Methods

### Sample Collection

*C*. *elegans* was grown according to standard procedures [48]. *G*. *pallida* were collected as described [49]. *T*. *spiralis* adults and L1 were isolated according to standard procedures. *N*. *brasiliensis* adults were isolated from rodents and L1 larvae were collected from fecal matter according to standard procedures. *E*. *brevis* were collected from soil filtrates in Sylt, Germany with assistance from Werner Armonies (Alfred Wegener Research Station) and *O*. *rectangula* were collected from sand filtrates in Vancouver, Canada. In both cases collected nematodes were stored for transport in 1 ml RNA Later. *P*. *varius* were collected by placing infected crickets in water and frozen at −80°C for RNA extraction.

### RNA Extraction and Small RNA Library Preparation

RNA was extracted using Trizol according to standard procedures. RNA Following RNA isolation, RNA was treated with 20U 5′ polyphosphatase (Epicenter) for 30 min to remove 5′ triphosphates and allow 5′ independent library construction or used directly for 5′ dependent library construction (S1 Fig.). To test for protection against 3′ end oxidation, we treated RNA resuspended in sodium borate buffer (pH 8.6) either with sodium periodate at a final concentration of 25 mM or with an equal volume of water as a control sample. We incubated for 10 min at room temperature and quenched the reaction with glycogen for 10 min before desalting the RNA with a G25 column (GE healthcare) and precipitating with Ethanol.

All small RNA libraries were made with the Illumina Truseq method according to the manufacturer’s instructions.

### Analysis of Small RNA Sequencing Data

Fasta files with one sequence for each read were generated by using Cutadapt (v1) to trim adapters and a custom Perl script to convert fastq into fasta files. Small RNAs of between 15 and 33 nt were then selected using a custom script written in Perl. In the absence of genome sequence, a custom script written in Perl was used to collect the length and first nucleotide of all species and tabulate this information. The output was read into R to generate barplots. To carry out alignments, to various genomes or transposon sequences from RepeatMasker predictions, we used Bowtie [50] with parameters—v 0—k 1 —best, to select only for reads that matched perfectly to the genome. This step generated sam files of the alignments, which were converted into bam files using samtools [51] and bed files using Bedtools [52] before reading into R for further analysis. Detailed description of the analysis of specific classes of small RNAs is available in S1 Text.

### Protein Evolution Analysis

In order to interrogate protein evolution we independently developed a method similar to those used for similar analyses [53]. To test for the conservation of *C*. *elegans* proteins across the nematode phylum, the predicted proteome from each species analysed was downloaded as a fasta file from the sources indicated in S1 Text. As a non-nematode species we downloaded the predicted *D*. *melanogaster* proteome from Flybase (www.flybase.org). Formatdb was then run on each species separately to create individual databases. The *C*. *elegans* predicted proteome was used as input and blastp was run separately against each database. This generated output files that were filtered using a custom Perl script running in the BioPerl environment to select the best blast hit for each *C*. *elegans* protein for each database tested. In order to select the best hit, the bit score was divided by the length of the input sequence, as this method is more reliable than using the e-value [54]. To draw the heatmap in Fig. 2, a curated list of small RNA related proteins was selected from the complete table. In order to test the statistical significance of the incongruity of the PRG-1 loss to the rest of the proteome we replaced the conservation table with a binary matrix representing presence absence calls at the 0.18 threshold, which is average length normalized bit score seen for PRG-1 in non-clade V nematodes. We then used a Jack-knife method to test how likely the pattern of conservation seen in PRG-1 is to occur amongst the proteome. We randomly removed 10% of the data and counted the number of proteins remaining above this threshold in each species, and repeated this to give 10,000 total jack-knifes, and the number of times that *D*. *melanogaster* appeared to have lost fewer proteins than all other non-clade V nematodes. This did not occur once in any of the simulations, thus giving an estimated *p*-value of <1e−4. For identification of selected RNAi proteins in *E*. *brevis* and *H*. *dujardini*, transcriptome databases from the draft transcriptomes were built using Formatdb and tblastn was used with *C*. *elegans* proteins. The best hit for selected proteins was then tested by reciprocal blast against *C*. *elegans* proteins to test for orthology.

To construct phylogenetic trees of Piwi and RdRP proteins we used separate runs of phmmer with *D*. *melanogaster* Piwi. *C*. *elegans* RRF-1 and *C*. *elegans* RRF-3 to identify the best hit of each in the nematode genomes. The sequences were aligned using Muscle using 16 iterations and the alignment was refined with Gblocks 0.91b using default parameters. Phylogeny was obtained using PhyML using the Blosum62 substitution matrix [55].

### DNA Methylation Analysis

To test for DNA methylation in our strain of *T*. *spiralis*, which may have differences to the isolate used by Gao and colleagues [44], we purified genomic DNA from the interphase of a Trizol extraction and used the Bisulfite-gold kit to convert these. We used Taq PCR and the primers from Gao and colleagues [44] and sequenced the PCR products directly. This confirmed 50% methylation at the CG sites identified [44].

We took DNA methylated genes (including transposon coding sequences) as defined [44] and identified the number of small RNAs mapping to these genes and compared to all genes. To estimate the density of DNA methylation at transposons, we used a custom script written in Perl to extract all CHG and CG potential methylation sites within each repetitive element predicted by Repeatmasker and interrogated the methylation status. CHG methylation genome-wide was around 4-fold lower than CG methylation. We grouped repetitive sequences by classes and calculated the percent methylation at each site as the number of converted reads/total reads and then counted the number of bases showing greater than 5% methylation. A chi-squared test was used to calculate the significance using the genome-wide percentage methylation to predict the expected value.

## Supporting Information

S1 Data Multiple sequence alignment for PRG-1/Piwi homologues. (TXT)

S2 Data Multiple sequence alignment for RdRPs. (TXT)

S3 Data Nuclear localization sequence in *T*. *spiralis* Dicer. (DOCX)

S4 Data Processed data underlying figures and charts. (ZIP)

S1 Fig Small RNA sequencing methodology. (A) 5′ dependent library preparation only allows RNAs with 5′ monophosphates to be ligated to adapters. It thus allows sequencing of Dicer products (both miRNAs and siRNAs) and mature piRNAs.(B) 5′ independent library preparation allows both 5′ triphosphate and 5′ monophosphate species to be ligated to adapters and thus enables sequencing of RNA dependent RNA polymerase products (22G-RNAs in *C*. *elegans*).
(PDF)

S2 Fig Further analysis of conservation of miRNA sequences and regulation. (A) Mean miRNA conservation across eight nematode species is plotted against expression of the miRNA in adult *C*. *elegans*. (B–D) Developmental expression changes of miRNAs in nematode species compared to that of the homologous miRNA in *C*. *elegans*, with the Spearman’s rank correlation coefficient shown for each species.
(PDF)

S3 Fig Small RNA sequencing of putative nematode outgroups. (A) 5′ monophosphate small RNAs in the tardigrade *H*. *dujardini*, showing longer sequences with a 5′ U bias, putative piRNAs. (B) Putative *H*. *dujardini* piRNAs show a prominent ten nucleotide overlap, with the tenth nucleotide of the 5′-most piRNA showing a bias towards (A), consistent with ping-pong amplification. (C, D) 5′ monophosphate and 5′ triphosphate small RNA sequencing from *P*. *varius* (Nematomorpha) showing absence of longer 5′ U species and no evidence of 5′ triphosphorylated small RNAs.
(PDF)

S4 Fig Small RNA sequencing of clade V, clade II, and clade I nematodes confirms absence of 5′ triphosphorylated small RNA populations and piRNAs outside of clade V. (A, B) 5′ monophosphate only (A) and 5′ mono and triphosphate (B) sequencing of small RNAs from *P*. *pacificus* (clade V). (C) 5′ mono and triphosphate sequencing of small RNAs from *N*. *brasiliensis* (clade V). (D, E) 5′ monophosphate only (D) and 5′ mono and triphosphate (E) sequencing of small RNAs from *E*. *brevis* (clade II) collected from Sylt in Germany. (F) 5′ mono and triphosphate sequencing of small RNAs from *O*. *rectangula* (clade II) collected from Vancouver in Canada. (G, H) 5′ monophosphate only (G) and 5′ mono and triphosphate (H) sequencing of small RNAs from *R*. *culicivorax* (clade I). (I) Collapsing the *R*. *culicivorax* sequences so that only unique sequences are represented removes the prominent 26T peak; this represents one abundant sequence and is thus not likely to be a piRNA, consistent with the absence of PRG-1/Piwi in this species.
(PDF)

S5 Fig Supplemental analysis of the evolution of PRG-1 in nematodes. (A) Blastp score in bits/length for the best hit to *C*. *elegans* PRG-1 compared to the median and interquartile range of the best hit for all *C*. *elegans* proteins. Members of the Piwi subfamily are shown in red (see Fig. 2E) and members of the Ago subfamily shown in purple (see Fig. 2E). (B) Histogram showing the result of 1,000 simulations the evolution of PRG-1 to the distance between *C*. *elegans* PRG-1 and *D*. *melanogaster* Piwi. *x*Axis is the e-value found after spiking the evolved protein into the *T*. *spiralis* genome; the red line represents the e-value of the best hit to PRG-1 within the true *T*. *spiralis* genome.
(PDF)

S6 Fig Small RNA sequencing of transposon-matching 5′ triphosphorylated siRNAs in clade III and clade IV nematodes. (A) 5′ mono and 5′ triphosphate sequencing demonstrates that 22–26 nt triphosphorylated small RNAs align predominantly antisense to transposons in *G*. *pallida*. (B) Collapsing to unique sequences retains the bias towards antisense orientation in both *G*. *pallida* and *B*. *malayi*. (C, D) 5′ monophosphate only sequencing shows evidence of 23 nt 5′ monophosphate small RNAs aligning antisense to transposon sequences in both *B*. *malayi* (C) and *G*. *pallida* (D), indicating that Dicer recognises transposons in these organisms.
(PDF)

S7 Fig Potential mechanisms for generation of siRNAs from transposons by Dicer. (A) Dicer cleavage of dsRNA originating from transcription of transposon sequences could feed into the small RNA pathway. (B) RNA dependent RNA polymerase could generate long dsRNA using the transposon sequence as a template, which would then be processed by Dicer to generate siRNAs.
(PDF)

S8 Fig Further analysis of *T*. *spiralis* siRNAs. (A) Sequencing of small RNAs following treatment with 200 mM sodium periodate compared to control samples shows that *C*. *elegans* 21U-RNAs are specifically protected against oxidation whilst *T*. *spiralis* 23–25 nt siRNAs are lost following oxidation. The peak at 28–30 nt in *T*. *spiralis* reflects two abundant ribosomal RNA sequences as shown by its loss upon collapsing the sequence data to unique sequences (far right hand panel). (B, C) Cluster analysis across the genome shows that regions with high density of piRNAs found in *C*. *elegans* and *D*. *melanogaster* are not found for *T*. *spiralis* 23–25 nt siRNAs. (B) Shows genome-wide distribution of *T*. *spiralis* 23–25 nt siRNAs, *C*. *elegans* piRNAs, and *D*. *melanogaster* piRNAs. Reads are binned in 100 kb windows across the genome and coloured by contigs or chromosomes according to the genome assembly, with the contigs or chromosomes sorted in order of the total number of small RNAs mapping to them. (C) Shows the cumulative fraction of sequences in (B) that are found in regions with greater than or equal to the density indicated on the *x*-axis. *C*. *elegans* and *D*. *melanogaster* both have more sequences mapping to higher density regions than *T*. *spiralis* does.
(PDF)

S1 Text Supplementary tables 1 and 2 and extended experimental procedures. (DOCX)

## Abbreviations

dsRNA: double-stranded RNA
miRNA: micro RNA
nt: nucleotide
piRNA: Piwi interacting RNA
RdRP: RNA dependent RNA polymerase
RNAi: RNA interference
siRNA: small interfering RNA
