# Supplementary material for: Ancient and Novel Small RNA Pathways Compensate for the Loss of piRNAs in Multiple Independent Nematode Lineages
Source: PLoS Biol. 2015 Feb 10;13(2):e1002061. doi: 10.1371/journal.pbio.1002061 (PMC4323106; doi:10.1371/journal.pbio.1002061)
Supplement: S3 Data — (DOCX) [file pbio.1002061.s003.docx]

Trichinella spiralis nuclear Dicer

**MGMAASSTNGDAMEDESEMEAFDVVRIADDVIQRLRFGSQDIFKSEDPVI 50**

**DDMLIESAYRIQYETLRQDLIATQEEVARLKRQLQERDYAERKKGRSGTV 100**

**EVGTKIEKHCSRRENQKLSNELVKLLLILEFMFTVQYSCLSNSATFRKNN 150**

**IDMVYGTLNNLVRASKDCVTELERHVSQVLYDLKHSQDLQYYVKEDLTFV 200**

**SAKEIDVGNRAVIIVFVPFPQYKQYRRILVRLIHEVEKKFGGKHVVFVAK 250**

**RRILPKPTRGKRKITQKQKRPRSRTLAAVHDEYLNDIVFPAEIVGKRIRV 300**

**RLDGSRLLKVHLDRNQKAAVEHKVNRMEKEQSGRLENVRLYQKELLECAK 350**

**KENVIVTLGTGTGKTFIAVMLIREMSESVHQPLKEGGKRSLFIVDKVPLV 400**

**KQQAEHIRINTNLKVGEFHGALGVDCWSAKIWMEFFDKFHVLVMTAEIFR 450**

**NILTHGFIKFDIVNLIVFDECHHATKQHPYKKIMELYKLCYNNNDSGIQP 500**

**RILGLTASVMNKKGDEIGLQKAIRNLETTLCSRVVGTSHAELLKLYSADP 550**

**HIAIVACKNDNKYDWHELDLFFQHLVRSTQKSLERCGRECEGNVSLYIQH 600**

**VCDVLAKLDSIMGQLGPWCALQVCKQMLQSTTNLATRISTLLADDEDNNL 650**

**FSLFTSGSDASSSVWLEVLKTCLKVTSVKLKRHIGLVDSVQKLKCVFIRQ 700**

**QFVDQQQQAVWNCIRQSTPGSLLYSGELNFQLEKNNSRKFENTNGANGPP 750**

**FQILIDTAKKLQPRSYSYLEVDYVVGSRMSAEVAEPSFARQEEVLKNFRH 800**

**GKLNLLAATSILEEGIDVRHCNYVIRFDTPLTFRSFVQSKGRARQKIAYY 850**

**TILVQDRFLESFQEMLNSFVETEKFLKVNGRDLNILTRTKDRDRSYSGNN 900**

**DQVDNCRKEVNLPNGDVDSLVEPYYTYFEENGLVKKAACLVLSSAHNVIK 950**

**RYCNKLRKDRFADSSPKFSVQTILNKDLSISYVATVQLPTTSPLKKKIEG 1000**

**KPMQNAKLAEMAAAFETAKMLHAMGELNEFLIPPVTVRKEIELKEDEQFF 1050**

**SISSRSYYLKEVPDALYKAIPRADQKSYLYAISISSIESFLPRLGILVSK 1100**

**PIGNLPGFSVFTDESYIDVEIEFVEETSYSAHQLDILTSFHCCIFKNYLF 1150**

**REEEDFIFDPENAACSYLIVPLKCEYNSKCSVDLSTAEKIINWSKDVSHI 1200**

**PQQRSEKFIMNSSSYFNAVVYPWYKNKNDRDFYYFVTAVDNTCTPMSPFP 1250**

**LKMYRSFAEYFEVTKQVKVLDKNQPLLSVKMVSFKRLNLLCEKPISILLF 1300**

**DFDDDANSCHCKLVPELVIIHPMPASMWRCLIFLPTVLYRMNHLLIAEQL 1350**

**RLQILREAMFPGEVLEENCEIQPLNKDWYLLASRLNDMCIVDNQLPVKLS 1400**

**KLSVNSNSAAAATATGSIDPETQALLEEFDVRFCNACPTVPVDDQLDILT 1450**

**GNSVAESHTAESNDECLFDTHFSLQLLSSIQPQQQPLEKVDFGIVESDGE 1500**

**SDFFVAAPALPESELNLFVEPSDVSDTATAPAQHRPGPQCRDVLRALTLR 1550**

**KAQDMFDLESMEALGDSFLKFIVSLHVFIKETNWNEGRLTSLRSEIVSNT 1600**

**NLFNLGKQKLLQAKLTAVPFDPTAQWLPPCFRSLAALESGYESVNELIDE 1650**

**GDSKKKNEALKANTPTPVVVAERYEMCSLNRTHQVIYDKSIADCVEALVG 1700**

**CYLLEAGMRPAIKLLKWFGIDIDGNLMNLLSSSSSSSSSSSTAFCSENCV 1750**

**LIGPEAKIHSIWTAYDLNSFEAKIGYRFTNKAYLIQALTHSSYNEVETPV 1800**

**TDSYERLEFLGDAILDYLISRHLYSSKRIRSPGLLSDLRAALVNNYFLYF 1850**

**NAELLSVNERFVLAMRGLKESVNFHNELYMMEEEQDDDEKETSEFAEHVE 1900**

**VPKPLGDIFESVAGAIFLDSHCSLATVWQVYYNMIAEEIGKQRSDFTSFN 1950**

**KCLCHPPISPVRHLLELEPERVQFNILDREEGNSGVHVQVVVTGKGSFIG 2000**

**SGKSYRTAKHAAAKKAVRELYPTSNHFII                      2029**

**For comparison Spombe**

|  |
| --- |
| **MDISSFLLPQLLRKYQQDVYNIASKQNTLLVMRTGAGKTLLAVKLIKQKL 50**  **EEQILIQESNLEHKKISVFLVNKVPLVFQQAEYIRSQLPAKVGMFYGELS 100**  **IEMSEQLLTNIILKYNVIVITADLFYLFLARGFLSINDLNLIIFDECHHA 150**  **IGNDAYARIMNDFYHRAKAVLSKKHFTLPRIFGMTASPFTGKKGNLYHRL 200**  **YQWEQLFDSKAHVVSENELADYFCLPEESYVMYSNKLVVPPSDSIIKKCE 250**  **ETLQGCKLISRAVKTALAETIDMGLWFGEQVWLYLVDFVETKRLKKKALG 300**  **KQLSDDEELAIDRLKIFVEDWKNNKYSDNGPRIPVFDSTDVTDKVFKLLE 350**  **LLKATYRKSDSVRTVIFVERKATAFTLSLFMKTLNLPNIRAHSFIGHGPS 400**  **DQGEFSMTFRRQKDTLHKFKTGKYNVLIATAVAEEGIDVPSCNLVIRFNI 450**  **CRTVTQYVQSRGRARAMASKFLIFLNTEELLIHERILHEEKNLKFALSEL 500**  **SNSNIFDSLVCEERERVTDDIVYEVGETGALLTGLYAVSLLYNFCNTLSR 550**  **DVYTRYYPTFTAQPCLSGWYCFEVELPKACKVPAAQGSPAKSIRKAKQNA 600**  **AFIMCLDLIRMGLIDKHLKPLDFRRKIADLETLEEDELKDEGYIETYERY 650**  **VPKSWMKVPEDITRCFVSLLYTDANEGDNHIFHPLVFVQAHSFPKIDSFI 700**  **LNSTVGPRVKIVLETIEDSFKIDSHLLELLKKSTRYLLQFGLSTSLEQQI 750**  **PTPYWLAPLNLSCTDYRFLENLIDVDTIQNFFKLPEPVQNVTDLQSDTVL 800**  **LVNPQSIYEQYAFEGFVNSEFMIPAKKKDKAPSALCKKLPLRLNYSLWGN 850**  **RAKSIPKSQQVRSFYINDLYILPVSRHLKNSALLIPSILYHIENLLVASS 900**  **FIEHFRLDCKIDTACQALTSAESQLNFDYDRLEFYGDCFLKLGASITVFL 950**  **KFPDTQEYQLHFNRKKIISNCNLYKVAIDCELPKYALSTPLEIRHWCPYG 1000**  **FQKSTSDKCRYAVLQKLSVKRIADMVEASIGACLLDSGLDSALKICKSLS 1050**  **VGLLDISNWDEWNNYFDLNTYADSLRNVQFPYSSYIEETIGYSFKNKKLL 1100**  **HLAFIHPSMMSQQGIYENYQQLEFLGDAVLDYIIVQYLYKKYPNATSGEL 1150**  **TDYKSFYVCNKSLSYIGFVLNLHKYIQHESAAMCDAIFEYQELIEAFRET 1200**  **ASENPWFWFEIDSPKFISDTLEAMICAIFLDSGFSLQSLQFVLPLFLNSL 1250**  **GDATHTKAKGDIEHKVYQLLKDQGCEDFGTKCVIEEVKSSHKTLLNTELH 1300**  **LTKYYGFSFFRHGNIVAYGKSRKVANAKYIMKQRLLKLLEDKSNLLLYSC 1350**  **NCKFSKKKPSDEQIKGDGKVKSLT** |

**Dicer-like 4 (Arabidopsis)**

**MRDEVDLSLTIPSKLLGKRDREQKNCEEEKNKNKKAKKQQKDPILLHTSA 50**

**ATHKFLPPPLTMPYSEIGDDLRSLDFDHADVSSDLHLTSSSSVSSFSSSS 100**

**SSLFSAAGTDDPSPKMEKDPRKIARRYQVELCKKATEENVIVYLGTGCGK 150**

**THIAVMLIYELGHLVLSPKKSVCIFLAPTVALVEQQAKVIADSVNFKVAI 200**

**HCGGKRIVKSHSEWEREIAANEVLVMTPQILLHNLQHCFIKMECISLLIF 250**

**DECHHAQQQSNHPYAEIMKVFYKSESLQRPRIFGMTASPVVGKGSFQSEN 300**

**LSKSINSLENLLNAKVYSVESNVQLDGFVSSPLVKVYYYRSALSDASQST 350**

**IRYENMLEDIKQRCLASLKLLIDTHQTQTLLSMKRLLKRSHDNLIYTLLN 400**

**LGLWGAIQAAKIQLNSDHNVQDEPVGKNPKSKICDTYLSMAAEALSSGVA 450**

**KDENASDLLSLAALKEPLFSRKLVQLIKILSVFRLEPHMKCIIFVNRIVT 500**

**ARTLSCILNNLELLRSWKSDFLVGLSSGLKSMSRRSMETILKRFQSKELN 550**

**LLVATKVGEEGLDIQTCCLVIRYDLPETVTSFIQSRGRARMPQSEYAFLV 600**

**DSGNEKEMDLIENFKVNEDRMNLEITYRSSEETCPRLDEELYKVHETGAC 650**

**ISGGSSISLLYKYCSRLPHDEFFQPKPEFQFKPVDEFGGTICRITLPANA 700**

**PISEIESSLLPSTEAAKKDACLKAVHELHNLGVLNDFLLPDSKDEIEDEL 750**

**SDDEFDFDNIKGEGCSRGDLYEMRVPVLFKQKWDPSTSCVNLHSYYIMFV 800**

**PHPADRIYKKFGFFMKSPLPVEAETMDIDLHLAHQRSVSVKIFPSGVTEF 850**

**DNDEIRLAELFQEIALKVLFERGELIPDFVPLELQDSSRTSKSTFYLLLP 900**

**LCLHDGESVISVDWVTIRNCLSSPIFKTPSVLVEDIFPPSGSHLKLANGC 950**

**WNIDDVKNSLVFTTYSKQFYFVADICHGRNGFSPVKESSTKSHVESIYKL 1000**

**YGVELKHPAQPLLRVKPLCHVRNLLHNRMQTNLEPQELDEYFIEIPPELS 1050**

**HLKIKGLSKDIGSSLSLLPSIMHRMENLLVAIELKHVLSASIPEIAEVSG 1100**

**HRVLEALTTEKCHERLSLERLEVLGDAFLKFAVSRHLFLHHDSLDEGELT 1150**

**RRRSNVVNNSNLCRLAIKKNLQVYIRDQALDPTQFFAFGHPCRVTCDEVA 1200**

**SKEVHSLNRDLGILESNTGEIRCSKGHHWLYKKTIADVVEALVGAFLVDS 1250**

**GFKGAVKFLKWIGVNVDFESLQVQDACIASRRYLPLTTRNNLETLENQLD 1300**

**YKFLHKGLLVQAFIHPSYNRHGGGCYQRLEFLGDAVLDYLMTSYFFTVFP 1350**

**KLKPGQLTDLRSLSVNNEALANVAVSFSLKRFLFCESIYLHEVIEDYTNF 1400**

**LASSPLASGQSEGPRCPKVLGDLVESCLGALFLDCGFNLNHVWTMMLSFL 1450**

**DPVKNLSNLQISPIKELIELCQSYKWDREISATKKDGAFTVELKVTKNGC 1500**

**CLTVSATGRNKREGTKKAAQLMITNLKAHENITTSHPLEDVLKNGIRNEA 1550**

**KLIGYNEDPIDVVDLVGLDVENLNILETFGGNSERSSSYVIRRGLPQAPS 1600**

**KTEDRLPQKAIIKAGGPSSKTAKSLLHETCVANCWKPPHFECCEEEGPGH 1650**

**LKSFVYKVILEVEDAPNMTLECYGEARATKKGAAEHAAQAAIWCLKHSGF 1700**

**LC**
