# Supplementary figures and images for: Ancient and Novel Small RNA Pathways Compensate for the Loss of piRNAs in Multiple Independent Nematode Lineages
Source: PLoS Biol. 2015 Feb 10;13(2):e1002061. doi: 10.1371/journal.pbio.1002061 (PMC4323106; doi:10.1371/journal.pbio.1002061)

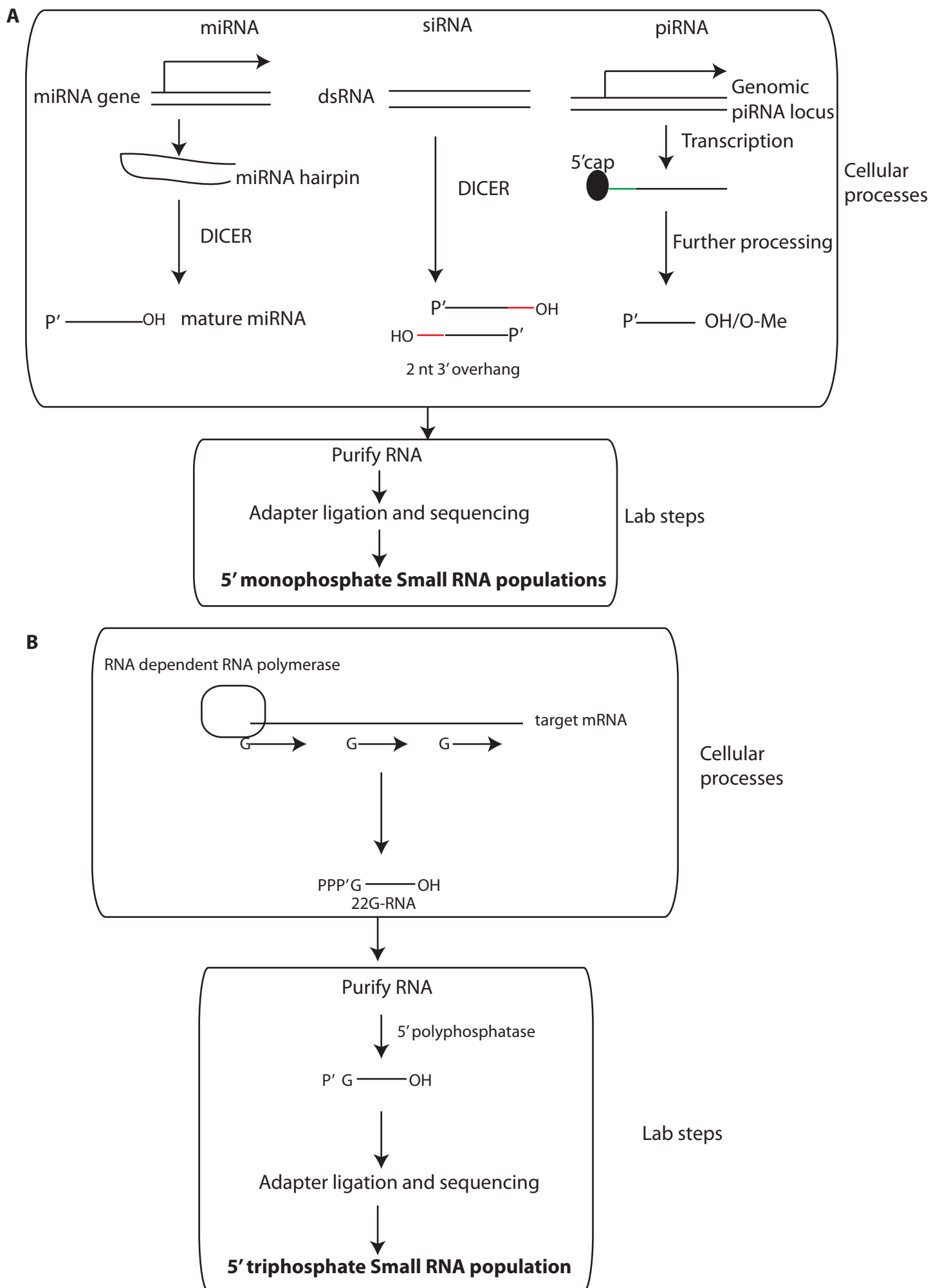

Supplemental Figure 1

Supplement: S1 Fig — (A) 5′ dependent library preparation only allows RNAs with 5′ monophosphates to be ligated to adapters. It thus allows sequencing of Dicer products (both miRNAs and siRNAs) and mature piRNAs.(B) 5′ independent library preparation allows both 5′ triphosphate and 5′ monophosphate species to be ligated to adapters and thus enables sequencing of RNA dependent RNA polymerase products (22G-RNAs in C. elegans). (PDF) [file pbio.1002061.s005.pdf]

**A**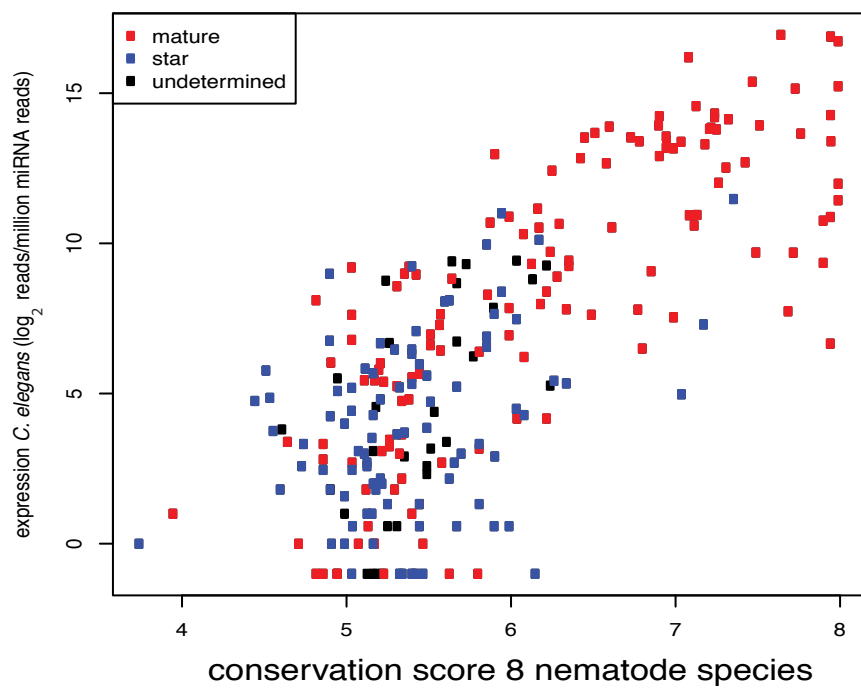**B**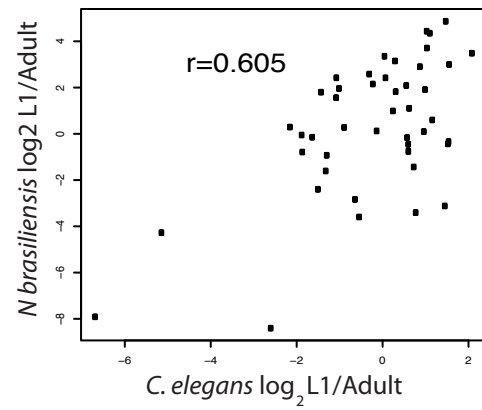**C**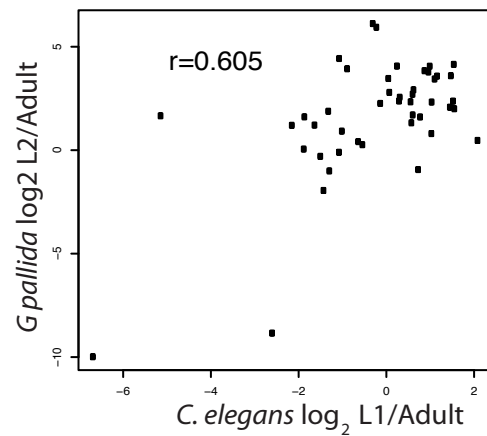**D**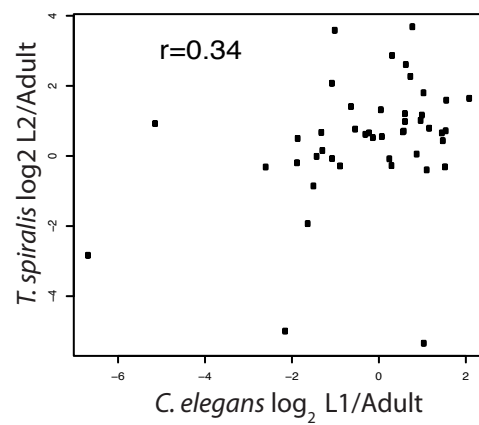

Supplemental Figure 2

Supplement: S2 Fig — (A) Mean miRNA conservation across eight nematode species is plotted against expression of the miRNA in adult C. elegans. (B–D) Developmental expression changes of miRNAs in nematode species compared to that of the homologous miRNA in C. elegans, with the Spearman’s rank correlation coefficient shown for each species. (PDF) [file pbio.1002061.s006.pdf]

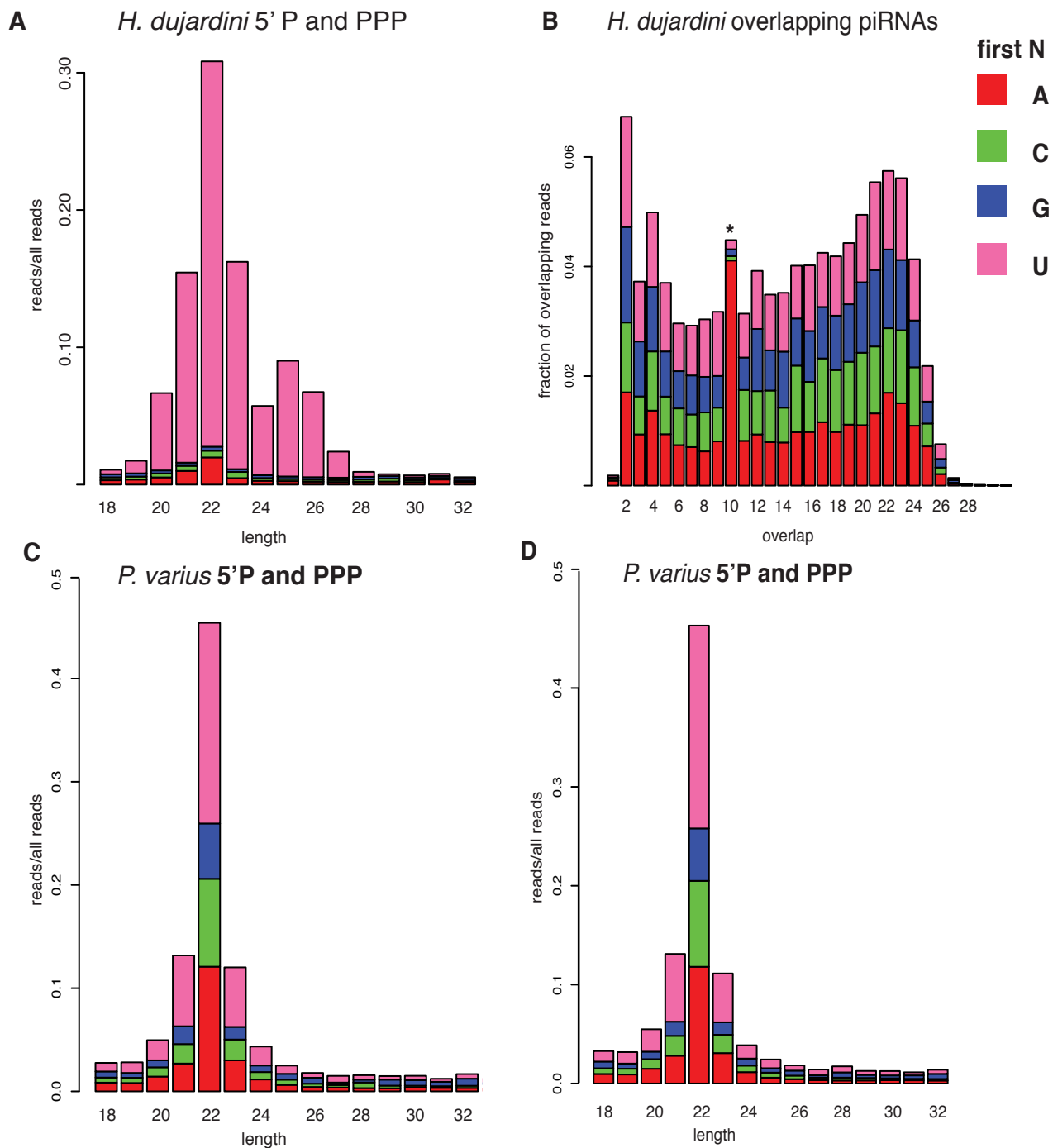

Supplemental Figure 3

Supplement: S3 Fig — (A) 5′ monophosphate small RNAs in the tardigrade H. dujardini, showing longer sequences with a 5′ U bias, putative piRNAs. (B) Putative H. dujardini piRNAs show a prominent ten nucleotide overlap, with the tenth nucleotide of the 5′-most piRNA showing a bias towards (A), consistent with ping-pong amplification. (C, D) 5′ monophosphate and 5′ triphosphate small RNA sequencing from P. varius (Nematomorpha) showing absence of longer 5′ U species and no evidence of 5′ triphosphorylated small RNAs. (PDF) [file pbio.1002061.s007.pdf]

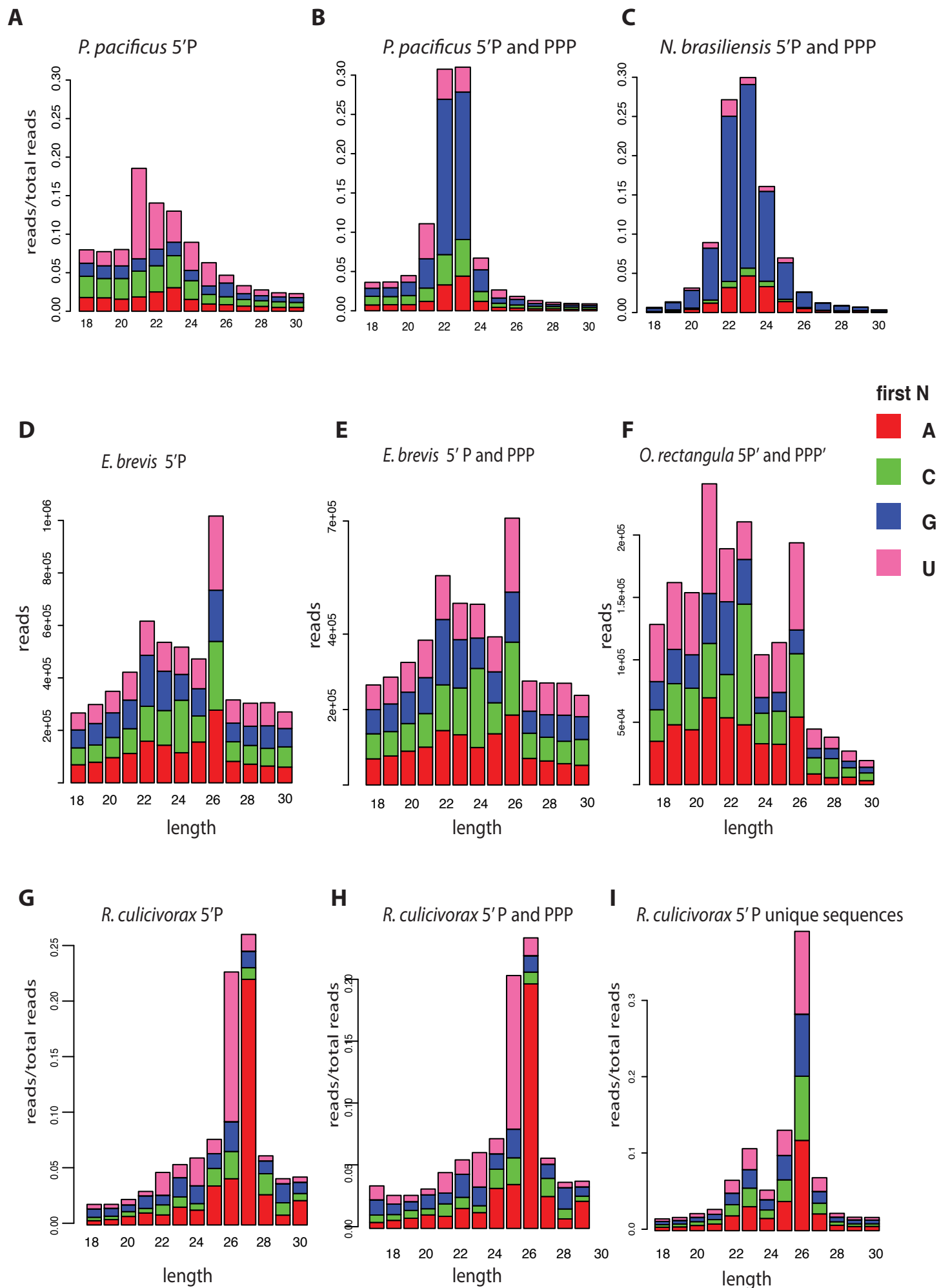

Supplemental Figure 4

Supplement: S4 Fig — (A, B) 5′ monophosphate only (A) and 5′ mono and triphosphate (B) sequencing of small RNAs from P. pacificus (clade V). (C) 5′ mono and triphosphate sequencing of small RNAs from N. brasiliensis (clade V). (D, E) 5′ monophosphate only (D) and 5′ mono and triphosphate (E) sequencing of small RNAs from E. brevis (clade II) collected from Sylt in Germany. (F) 5′ mono and triphosphate sequencing of small RNAs from O. rectangula (clade II) collected from Vancouver in Canada. (G, H) 5′ monophosphate only (G) and 5′ mono and triphosphate (H) sequencing of small RNAs from R. culicivorax (clade I). (I) Collapsing the R. culicivorax sequences so that only unique sequences are represented removes the prominent 26T peak; this represents one abundant sequence and is thus not likely to be a piRNA, consistent with the absence of PRG-1/Piwi in this species. (PDF) [file pbio.1002061.s008.pdf]

A

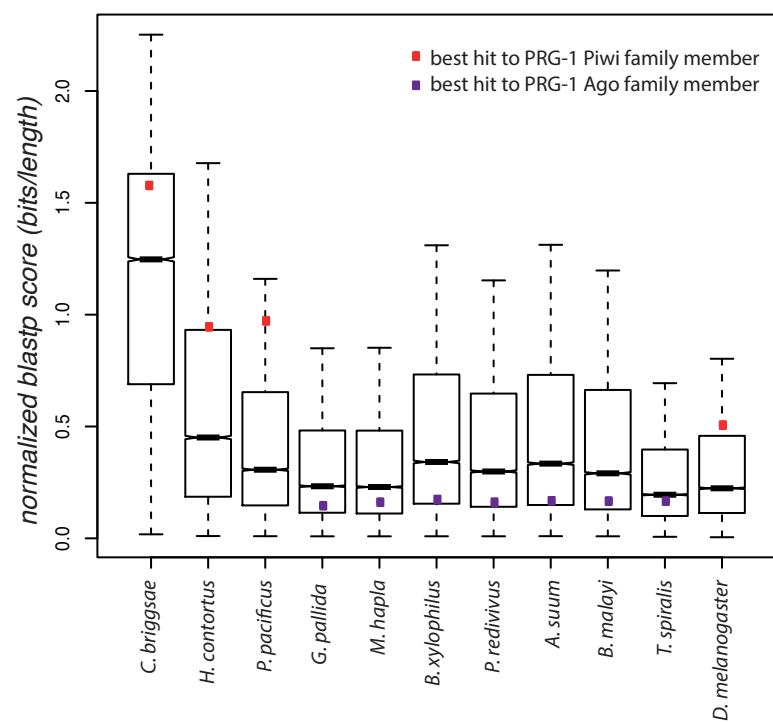

B

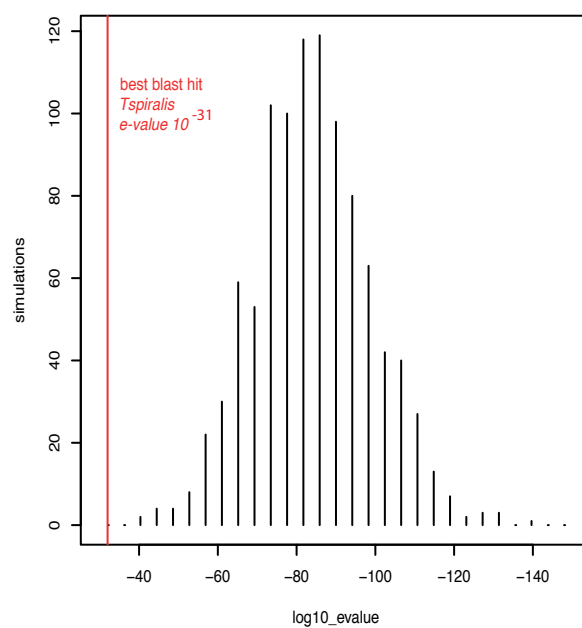

Supplemental Figure 5

Supplement: S5 Fig — (A) Blastp score in bits/length for the best hit to C. elegans PRG-1 compared to the median and interquartile range of the best hit for all C. elegans proteins. Members of the Piwi subfamily are shown in red (see Fig. 2E) and members of the Ago subfamily shown in purple (see Fig. 2E). (B) Histogram showing the result of 1,000 simulations the evolution of PRG-1 to the distance between C. elegans PRG-1 and D. melanogaster Piwi. xAxis is the e-value found after spiking the evolved protein into the T. spiralis genome; the red line represents the e-value of the best hit to PRG-1 within the true T. spiralis genome. (PDF) [file pbio.1002061.s009.pdf]

A

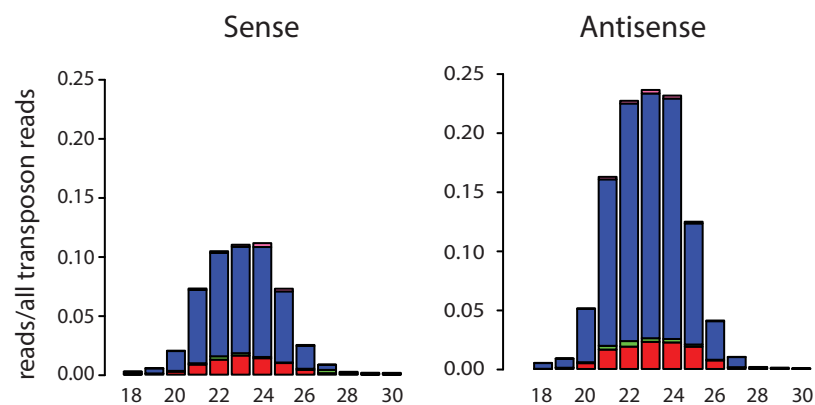

B

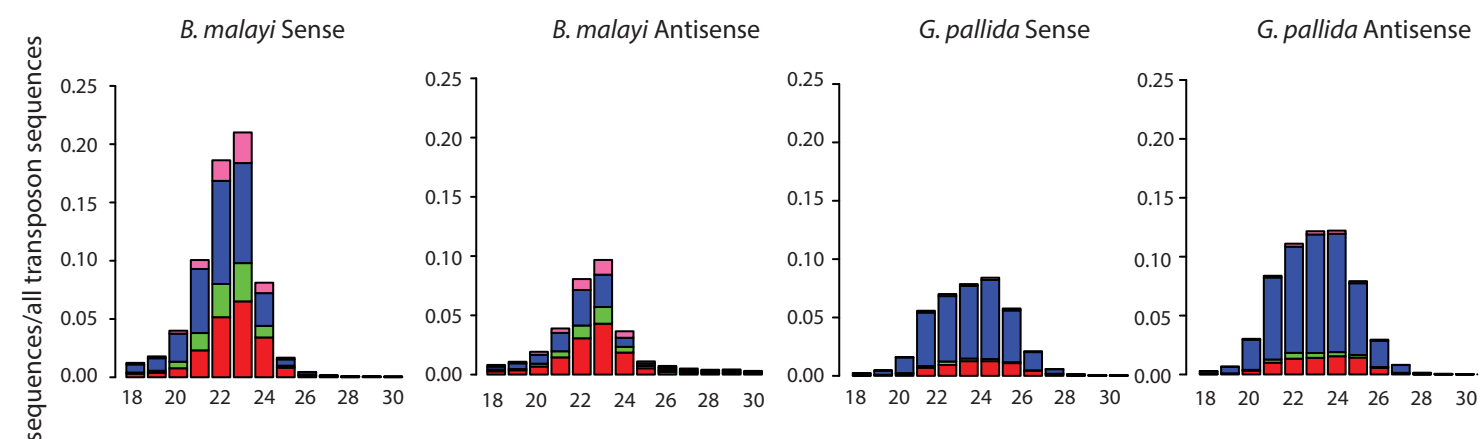

C

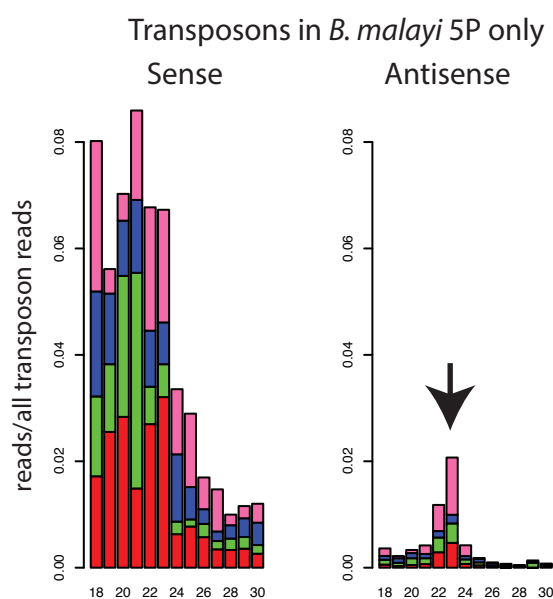

D

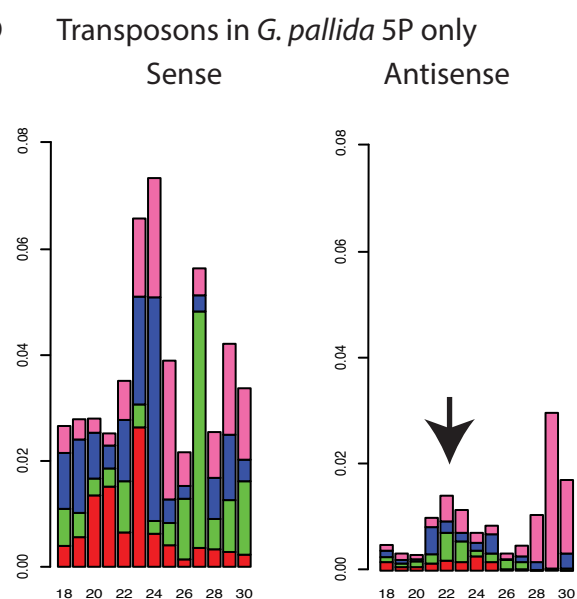

Supplemental Figure 6

Supplement: S6 Fig — (A) 5′ mono and 5′ triphosphate sequencing demonstrates that 22–26 nt triphosphorylated small RNAs align predominantly antisense to transposons in G. pallida. (B) Collapsing to unique sequences retains the bias towards antisense orientation in both G. pallida and B. malayi. (C, D) 5′ monophosphate only sequencing shows evidence of 23 nt 5′ monophosphate small RNAs aligning antisense to transposon sequences in both B. malayi (C) and G. pallida (D), indicating that Dicer recognises transposons in these organisms. (PDF) [file pbio.1002061.s010.pdf]

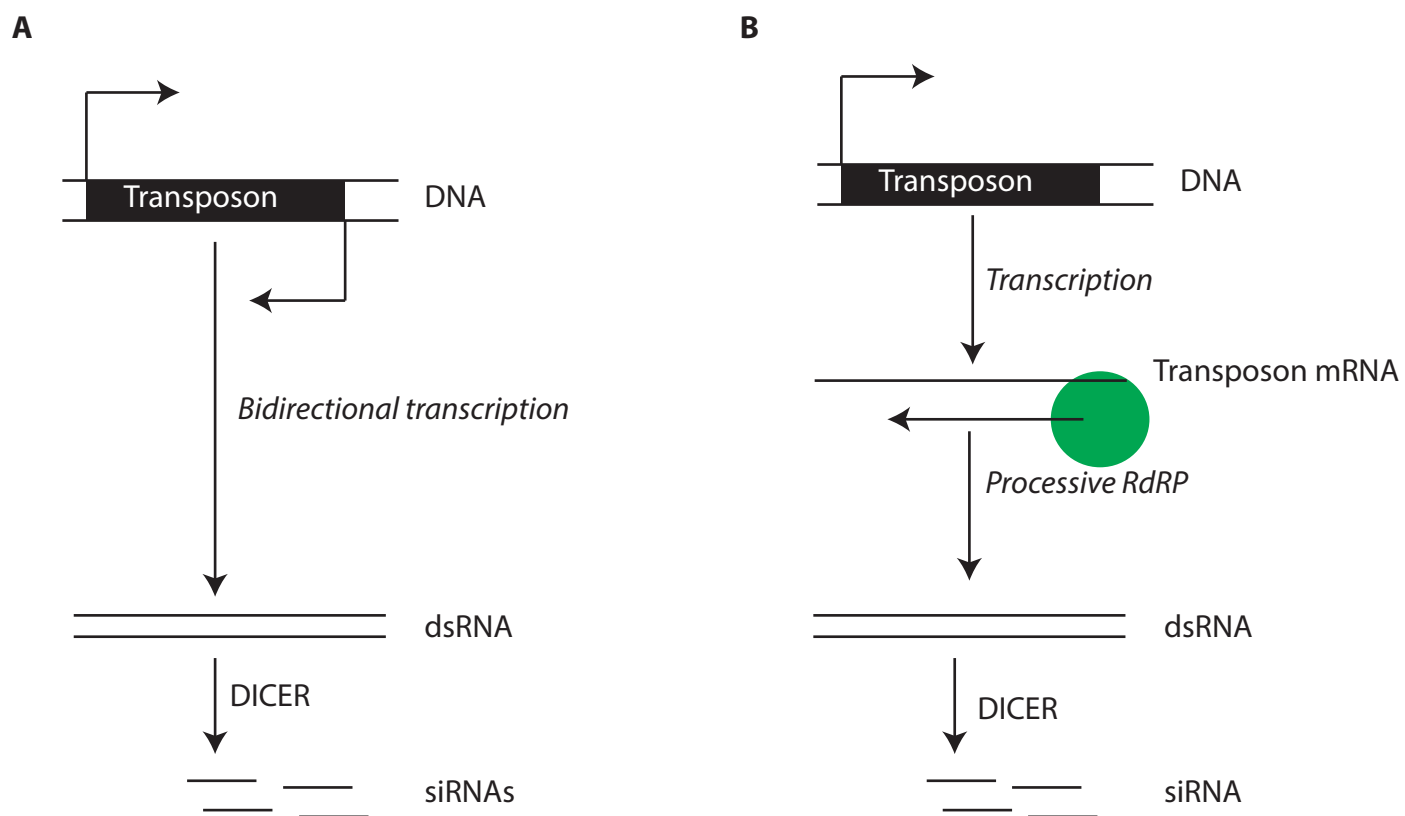

Supplemental Figure 7

Supplement: S7 Fig — (A) Dicer cleavage of dsRNA originating from transcription of transposon sequences could feed into the small RNA pathway. (B) RNA dependent RNA polymerase could generate long dsRNA using the transposon sequence as a template, which would then be processed by Dicer to generate siRNAs. (PDF) [file pbio.1002061.s011.pdf]

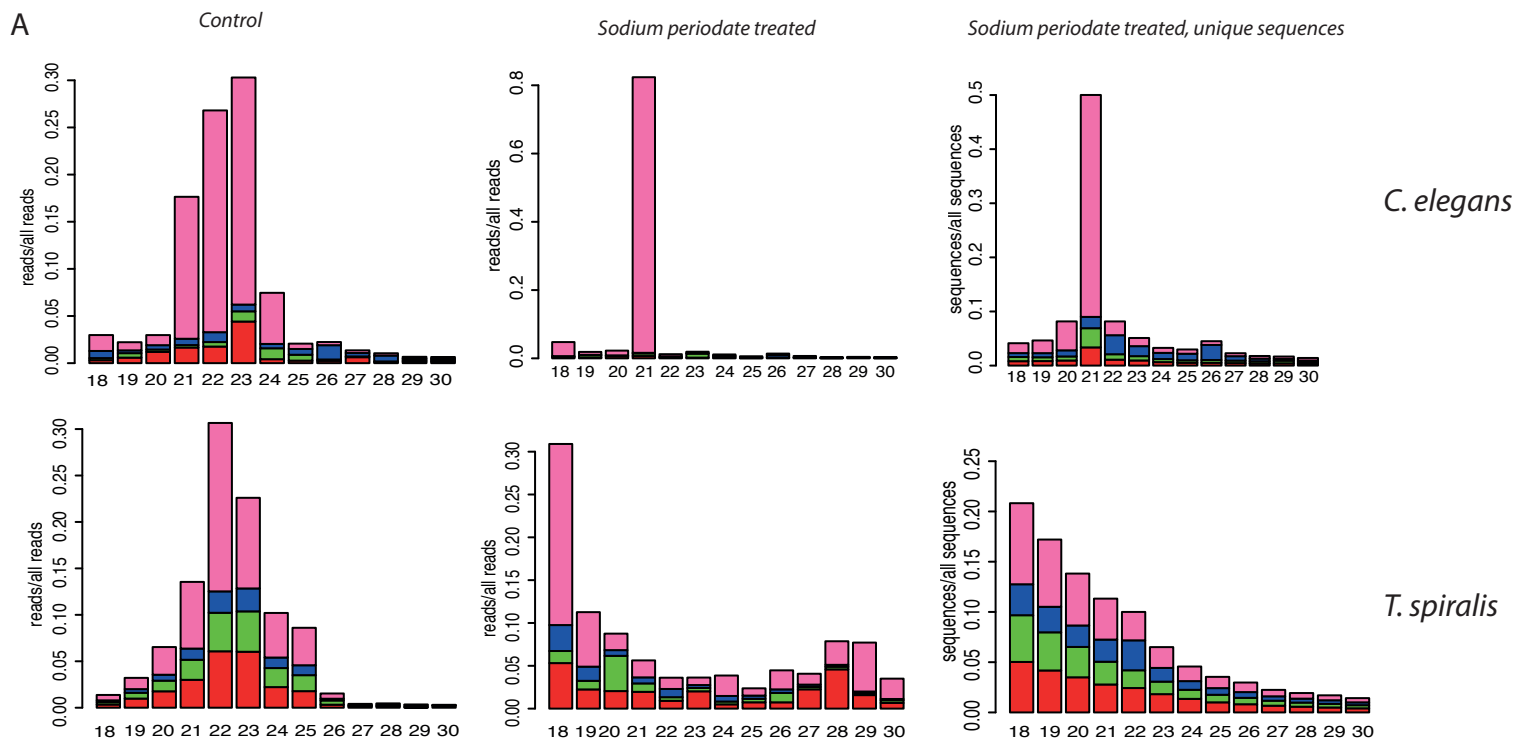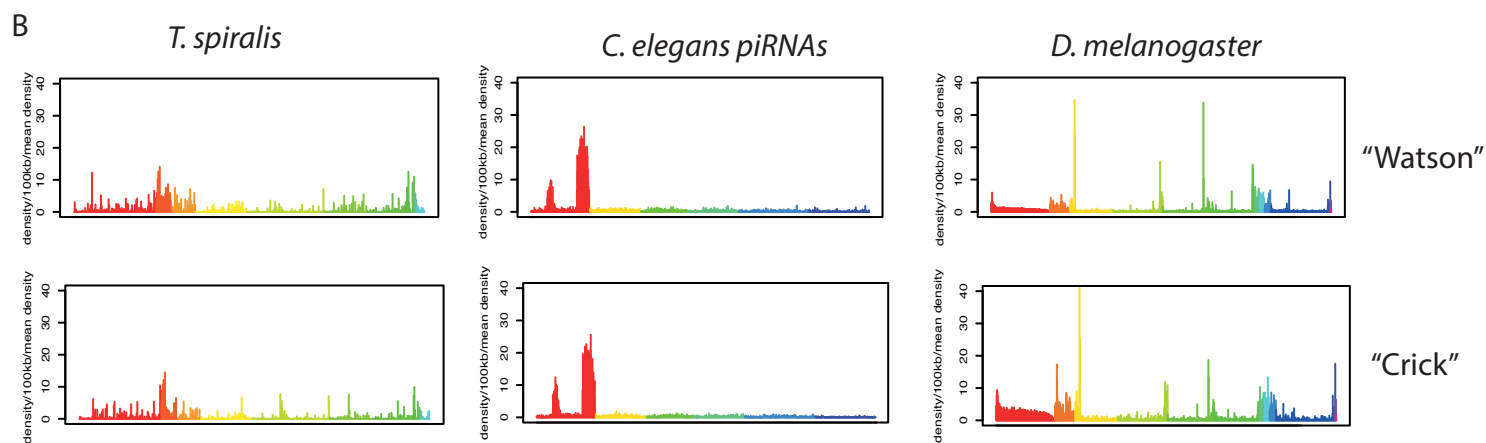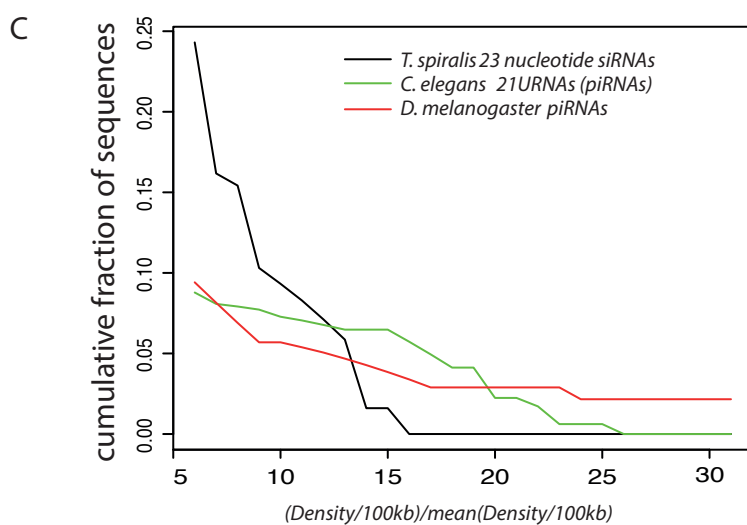

Supplemental Figure 8

Supplement: S8 Fig — (A) Sequencing of small RNAs following treatment with 200 mM sodium periodate compared to control samples shows that C. elegans 21U-RNAs are specifically protected against oxidation whilst T. spiralis 23–25 nt siRNAs are lost following oxidation. The peak at 28–30 nt in T. spiralis reflects two abundant ribosomal RNA sequences as shown by its loss upon collapsing the sequence data to unique sequences (far right hand panel). (B, C) Cluster analysis across the genome shows that regions with high density of piRNAs found in C. elegans and D. melanogaster are not found for T. spiralis 23–25 nt siRNAs. (B) Shows genome-wide distribution of T. spiralis 23–25 nt siRNAs, C. elegans piRNAs, and D. melanogaster piRNAs. Reads are binned in 100 kb windows across the genome and coloured by contigs or chromosomes according to the genome assembly, with the contigs or chromosomes sorted in order of the total number of small RNAs mapping to them. (C) Shows the cumulative fraction of sequences in (B) that are found in regions with greater than or equal to the density indicated on the x-axis. C. elegans and D. melanogaster both have more sequences mapping to higher density regions than T. spiralis does. (PDF) [file pbio.1002061.s012.pdf]
