# Supplementary material for: Ancient and Novel Small RNA Pathways Compensate for the Loss of piRNAs in Multiple Independent Nematode Lineages
Source: PLoS Biol. 2015 Feb 10;13(2):e1002061. doi: 10.1371/journal.pbio.1002061 (PMC4323106; doi:10.1371/journal.pbio.1002061)
Supplement: S1 Text — (DOCX) [file pbio.1002061.s013.docx]

**Supplemental materials**

**Supplemental Table 1: Nematode species investigated in this study**

**Supplemental Table 2: Reciprocal Blast analysis for selected *C. elegans* small RNA proteins against predicted proteins from additional species not shown in Figure 2.**

**Supplemental Methods**

**Supplemental References**

**Data S1: Alignment of RNA dependent RNA polymerases (muscle)**

**Data S2: Alignment of Piwi proteins (muscle)**

**Data S3: Identification of nuclear localization motif in *T. spiralis* Dicer.** Nuclear localization motifs were identified with [http://nls-mapper.iab.keio.ac.jp/](http://nls-mapper.iab.keio.ac.jp/cgi-bin/NLS_Mapper_y.cgi)

**Data S4: Processed data underlying graphs (zipped folder)**

**Supplemental Figures S1-S8**

**Supplemental Table 1**

| Species | Clade | Small RNA sequencing | Developmental stage | Genome sequence |
| --- | --- | --- | --- | --- |
| *Caenorhabditis elegans* | V | 5’P, 5’PPP | Adult, L1 | Wormbase WS236 |
| *Caenorhabditis briggsae* | V | *5’ PPP [1]* | Adult | Wormbase WS236 |
| *Pristionchus pacificus* | V | 5’ P, 5’PPP | Adult | Wormbase WS236 |
| *Nippostrongylus brasiliensis* | V | 5’P, 5’PPP | Adult, L1 | Unpublished; draft without gene models  http://www.sanger.ac.uk/research/initiatives/globalhealth/research/helminthgenomes/* |
| *Haemonchus contortus* | V | *5’P*[2] | Adult | Wormbase WS240[3,4] |
| *Globodera pallida* | IV | 5’P, 5’PPP | Adult, J2 | http://www.sanger.ac.uk/research/initiatives/globalhealth/research/helminthgenomes/[5] |
| *Meloidogyne hapla* | IV | N/A | N/A | Wormbase WS236 |
| *Panagrellus redivivus* | *IV* | *5’P [5]* | Adult | Wormbase WS240[6] |
| *Ascaris suum* | III | *5’ PPP*[7] | Adult | Wormbase WS236[7] |
| *Brugia malayi* | III | 5’ PPP, 5’P | Adult | Wormbase WS236 |
| *Enoplus brevis (Sylt)* | II | 5’P, 5’PPP | Adult | Draft transcriptome available at http://locifera.bio.ed.ac.uk/Enoplus_brevis |
| *Odontophora rectangula* | II | 5’PPP | Adult | NA |
| *Romanomeris culicivorax* | I | 5’P, 5’PPP | Adult | http://www.nematodes.org/genomes/romanomermis_culicivorax/[8] |
| *Trichinella spiralis* | I | 5’P, 5’PPP | L1(muscle), Adult | Wormbase WS236 |
| *Hypsibius dujardini*  *Tardigrade* | NA | 5’P, 5’PPP | Mixed | Boothby, T., Goldstein, B., (Personal Communication) AND  http://badger.bio.ed.ac.uk/H_dujardini/ |
| *Paragordius varius*  *Nematomorph* | NA | 5’P, 5’PPP | Adult | Draft transcriptome from Nembase (under preparation) |

Permission to use unpublished data granted by Matt Berrimann, Wellcome Trust Sanger Institute, Hinxton, Cambridge, UK.

**Supplemental Table 2**

| *C. elegans* protein | Best hit ID | Blast e-value | Reciprocal blast best hit | Reciprocal best hit e-value | Clade | Species |
| --- | --- | --- | --- | --- | --- | --- |
| PRG-1 | nDi.2.2.2.t04764 | 7.00E-34 | ALG-1 | 1.00E-200 | III | *D. immitis* |
| RRF-1 | nDi.2.2.2.t01275 | 1.00E-200 | RRF-1 | 1.00E-200 | III | *D. immitis* |
| RRF-3 | nDi.2.2.2.t06326 | 1.00E-200 | RRF-3 | 1.00E-200 | III | *D. immitis* |
| PRG-1 | EFO21055.1 | 1.00E-32 | ALG-1 | 1.00E-200 | III | *L. loa* |
| RRF-1 | EJD75145.1 | 1.00E-200 | RRF-1 | 1.00E-200 | III | *L. loa* |
| RRF-3 | EFO21905.2 | 1.00E-200 | RRF-3 | 1.00E-200 | III | *L. loa* |
| PRG-1 | Minc06774 | 2.00E-31 | ALG-1 | 1.00E-200 | IV | *M. incognita* |
| RRF-1 | Minc10000 | 1.00E-200 | RRF-1 | 1.00E-200 | IV | *M. incognita* |
| RRF-3 | Minc10000 | 3.00E-148 | RRF-1 | 1.00E-200 | IV | *M. incognita* |
| PRG-1 | OVOC2252 | 8.00E-34 | ALG-1 | 1.00E-200 | III | *O. volvulus* |
| RRF-1 | OVOC12047 | 1.00E-200 | RRF-1 | 1.00E-200 | III | *O. volvulus* |
| RRF-3 | OVOC3422 | 1.00E-200 | RRF-3 | 1.00E-200 | III | *O. volvulus* |
| PRG-1 | TMUE_s0141002500 | 2e-36 | ALG-1 | 1e-200 | I | *T. muris* |
| RRF-3 | TMUE_s0004001600 | 4e-167 | RRF-3 | 8e-162 | I | *T. muris* |
| RRF-1 | TMUE_s0004001600 | 2e-135 | RRF-3 | 8e-162 | I | *T. muris* |
| PRG-1 | isotig06070 | 2e-30 | ALG-3 | 5e-129 | I | *R. culicivorax** |
| RRF-3 | isotig11555 | 8e-111 | RRF-3 | 1e-111 | I | *R. culicivorax** |
| RRF-1 | isotig11555 | 1e-90 | RRF-3 | 1e-111 | I | *R. culicivorax** |
| PRG-1 | comp219419_c0_seq4 | 2e-32 | ALG-1 | 1e-165 | II | *E. brevis** |
| RRF-3 | comp220889_c0_seq1 | <1e-200 | RRF-3 | 1e-200 | II | *E.brevis** |
| RRF-1 | comp220889_c0_seq1 | 1e-156 | RRF-3 | 1e-200 | II | *E. brevis** |
| PRG-1 | comp60743_c0_seq1 | 2e-29 | ALG-1 | 1e-200 | *NA* | *P. varius** |
| RRF-3 | No hits ^ | NA | NA | NA | *NA* | *P. varius** |
| RRF-1 | No hits ^ | NA | NA | NA | *NA* | *P. varius** |
| PRG-1 | comp115314_c0_seq8 | 1e-101 | PRG-1 | 1e-101 | *NA* | *H. dujardini** |
| RRF-3 | No hits ^ | NA | NA | NA | *NA* | *H. dujardini** |
| RRF-1 | No hits ^ | NA | NA | NA | *NA* | *H. dujardini** |

^cut-off e-value 1e-3

*values are from tblastn and blastx using transcriptome datasets.

**Extended Experimental Procedures**

**Small RNA sequencing analysis**

*miRNA analysis*

To identify the homologues of miRNAs in different nematode species, we initially used miRDeep2[9] using *C. elegans* miRbase[10] miRNAs as a base species to identify predicted miRNAs on the basis of the ability of the genomic region surrounding the predicted mature miRNA sequence to form a hairpin, the presence of both mature and star reads and the absence of reads matching to predicted loop regions[9]. This allowed us to identify several novel miRNAs and conserved miRNAs in nematode species. However, the performance of miRDeep2 depends strongly on having as complete a genomic sequence as possible and we found that many highly conserved miRNAs that we could clearly identify as present in the species we tested were not predicted by miRDeep2. We therefore extended our methodology to identify miRNAs using a custom tool written in Perl that identified potential miRNAs through the conservation of the seed region (positions 2-8 in the mature sequence)[11] using all the miRNAs contained in miRbase as a reference. In this algorithm, if a small RNA was found to have the same seed as a miRbase miRNA, the total percentage identity across the entire sequence was calculated and recorded. After going through the entire miRbase database, the reference miRNA with the highest potential match across the entire sequence was outputted- in the case that multiple miRNAs with the same percentage identity were found these were all outputted. We used a cut-off of 60% of perfect identity across the entire sequence to define potentially homologous miRNAs. To analyse conservation of miRNAs with *C. elegans* miRNAs (Figure 1B) we selected all the miRbase *C. elegans* miRNAs for which we could find at least 50 reads in our *C. elegans* small RNA sequencing library. We defined mature sequences on the basis that they had at least 2-fold more reads than the star sequence and selected only the mature sequence for further analysis. Only one abundant miRNA (miR-46) did not indicate mature and star sequences on this basis and so we used both. We defined a conservation score for the miRNAs in all nematode species using the expression normalized to all predicted miRNAs relative to *C. elegans* and the conservation, using the following formula:

Conservation score = log2(|(R_species_ – R _C. elegans_)|/R _C. elegans_)+log2(I_species_+1)

Where R is reads/million and I is fractional identity (%identity/100).

The three sets of conservation drawn on Figure 1B are values of 0, -1 and -2 (decreasing stringency).

To analyse the presence or absence of conserved miRNA families we looked for miRNAs with the same seed that were conserved in *Drosophila* *melanogaster* and *Homo sapiens* having downloaded the mature miRNA sequences from miRbase (www.mirbase.org)[10]. We named these according to the most abundant member of the family in *C. elegans* if a *C. elegans* homologue existed or the human sequence if *C. elegans* was missing this miRNA. We then searched for these miRNA families, and calculated the conservation as the highest percentage identity to any member of the family.

*piRNA sequence analysis*

For analysis of the conservation of the upstream sequence motif in Clade V nematodes we used custom scripts written in BioPerl to extract 57bp upstream and 2 bp downstream of the start site of 21U RNAs that aligned perfectly to the genome sequence. We then used this as input to MEME using default parameters. The best predicted motif is shown.

For identification of potential ping-pong amplification in Tardigrades we selected all small RNAs 25 nucleotides or longer in the small RNA sequencing data from *H. dujardini* and aligned these to the genome. We searched for small RNAs that aligned to opposite strands. For all pairs we then tabulated the position of the overlap the nucleotide at the overlap position.

*Small RNAs mapping to transposon sequences*

We ran Repeatmasker (http://www.repeatmasker.org) using “animalia” as the reference species to identify repeat elements in nematode genomes. We then extracted these sequences from the genomes using custom scripts written in BioPerl using the Bio::db::fasta module. We then used this to build genomes using Bowtie-build and carried out alignments as described above. To identify genome-wide signature of Dicer processing in *T. spiralis* we took all the small RNAs aligning to transposons that were 23 nucleotides long and originated from opposite strands. We then calculated the overlap between the pairs on opposite strands and tabulated this information. Dicer is expected to produce pairs that have 2 nucleotide overhang at the 3’ end.

**Supplemental References**

1. Shi Z, Montgomery TA, Qi Y, Ruvkun G (2013) High-throughput sequencing reveals extraordinary fluidity of miRNA, piRNA, and siRNA pathways in nematodes. Genome Res 23: 497–508. doi:10.1101/gr.149112.112.

2. Winter AD, Weir W, Hunt M, Berriman M, Gilleard JS, et al. (2012) Diversity in parasitic nematode genomes: the microRNAs of Brugia pahangi and Haemonchuscontortus are largely novel. BMC Genomics 13: 4. doi:10.1186/1471-2164-13-4.

3. Laing R, Kikuchi T, Martinelli A, Tsai IJ, Beech RN, et al. (2013) The genome and transcriptome of Haemonchus contortus, a key model parasite for drug andvaccine discovery. Genome Biology 14: R88. doi:10.1186/gb-2013-14-8-r88.

4. Schwarz EM, Korhonen PK, Campbell BE, Young ND, Jex AR, et al. (2013) The genome and developmental transcriptome of the strongylid nematode Haemonchus contortus. Genome Biology 14: R89. doi:10.1186/gb-2013-14-8-r89.

5. Cotton JA, Lilley CJ, Jones LM, Kikuchi T, Reid AJ, et al. (2014) The genome and life-stage specific transcriptomes of Globodera pallida elucidate key aspects of plantparasitism by a cyst nematode. 15: 1–17. doi:10.1186/gb-2014-15-3-r43.

6. Srinivasan J, Dillman AR, Macchietto MG, Heikkinen L, Lakso M, et al. (2013) The draft genome and transcriptome of Panagrellus redivivus are shaped by the harsh demands of a free-living lifestyle. Genetics 193: 1279–1295. Available: http://eutils.ncbi.nlm.nih.gov/entrez/eutils/elink.fcgi?dbfrom=pubmed&id=23410827&retmode=ref&cmd=prlinks.

7. Wang J, Czech B, Crunk A, Wallace A, Mitreva M, et al. (2011) Deep small RNA sequencing from the nematode Ascaris reveals conservation, functional diversification, and novel developmental profiles. Genome Res 21: 1462–1477. doi:10.1101/gr.121426.111.

8. Schiffer PH, Kroiher M, Kraus C, Koutsovoulos GD, Kumar S, et al. (2013) The genome of Romanomermis culicivorax: revealing fundamental changes in the core developmental genetic toolkit in Nematoda. BMC Genomics 14: 923. Available: http://eutils.ncbi.nlm.nih.gov/entrez/eutils/elink.fcgi?dbfrom=pubmed&id=24373391&retmode=ref&cmd=prlinks.

9. Friedlander MR, Mackowiak SD, Li N, Chen W, Rajewsky N (2011) miRDeep2 accurately identifies known and hundreds of novel microRNA genes in seven animal clades. Nucleic Acids Res 40: 37–52. doi:10.1093/nar/gkr688.

10. Kozomara A, Griffiths-Jones S (2010) miRBase: integrating microRNA annotation and deep-sequencing data. Nucleic Acids Res 39: D152–D157. doi:10.1093/nar/gkq1027.

11. Lewis BP, Shih I, Jones-Rhoades MW, Bartel DP (2003) Prediction of Mammalian MicroRNA Targets. Cell. Available: http://www.sciencedirect.com/science/article/pii/S0092867403010183.
